# Relationships between plant hormones, carbohydrate metabolism and excision-induced adventitious root formation of two contrasting hydrangea cultivars

**DOI:** 10.1007/s00425-026-05156-y

**Published:** 2026-09-14

**Authors:** Uwe Druege, Niklas Mey, Tobias Geißler

**Affiliations:** Erfurt Research Centre for Horticultural Crops (FGK), Erfurt University of Applied Sciences, Kühnhäuser Str. 101, 99090 Erfurt, Germany

**Keywords:** Abscisic acid, Auxin, Cuttings, Cytokinin, Root development, Sugars

## Abstract

**Main conclusion:**

Cytokinins and abscisic acid act as auxin antagonists during adventitious root induction, thereby limiting AR development depending on plant genotype and the distance to the wounding site.

**Abstract:**

This study investigated the role of plant hormones in the lower rooting capacity of cuttings of the *Hydrangea macrophylla* cultivar ‘Clarissa’ compared to ‘Caipirinha’, further considering interrelations to carbohydrates. At first, the rooting response to 1 min pulse treatments of the stem base with indole-3-acetic acid (IAA) was analyzed. Without IAA, rooting of ‘Clarissa’ lagged behind that of ‘Caipirinha’. Increasing IAA doses up to 50 mM particularly enhanced rooting in ‘Clarissa’ to the same level as ‘Caipirinha’. The number of adventitious roots was positively correlated with the length of the rooting zone, which was greater in ‘Caipirinha’ and was further enlarged by IAA application. Carbohydrate analysis indicated higher carbohydrate utilization in the 0–1 cm basal stem section compared to the 1–2.5 cm section above, which was further stimulated by IAA. Phytohormone profiling by LC-MS/MS in both stem base sections revealed similar dynamics for jasmonic acid, jasmonoyl-isoleucine, and IAA in both cultivars, but significantly higher cytokinin and abscisic acid (ABA) levels in ‘Clarissa’ compared to ‘Caipirinha’. ‘Clarissa’ revealed lower IAA/cytokinin and IAA/(cytokinin + ABA) ratios during the first 48 h after cutting excision compared to ‘Caipirinha’, while these ratios were higher in the basal 0–1 cm compared to the 1–2.5 cm stem section above. These findings provide new perspectives on hormonal crosstalk in adventitious rooting and support the conclusion that the lower rooting capacity and shorter rooting zone of ‘Clarissa’ is likely based on locally higher cytokinin and ABA levels that antagonize IAA during the induction phase.

**Supplementary Information:**

The online version contains supplementary material available at https://doi.org/10.1007/s00425-026-05156-y.

## Introduction

Hydrangea (*Hydrangea macrophylla* (Thunb.) Ser.) is a perennial woody shrub that is of high economic importance as an ornamental crop for the European market and is vegetatively propagated by the rooting of cuttings. This is based on adventitious root (AR) formation as a key developmental process that involves the reprogramming of specific responsive cells in the stem base near the wound and is triggered by two main stimuli: wounding and isolation from the donor plant (reviewed in Druege et al. 2016, 2019). Some hydrangea cultivars are difficult-to-root, and there is a growing demand for efficient propagation protocols to address rising energy and labor costs in Europe (Druege and Chamas 2026). Considering the increasing importance of sustainable plant propagation and the environmental risks associated with the production and use of chemical plant growth regulators, there is a need for a better understanding of the endogenous physiological control of AR formation in *H. macrophylla* as a basis for the development of smart, knowledge-based tools and protocols.

Adventitious root formation in cuttings involves sequential phases. It begins with the induction phase, during which specific AR source cells near the wound site are reprogrammed, followed by the initiation phase, which starts with the appearance of the first new cell clusters and ends with the formation of dome-shaped AR primordia, and concludes with the expression phase, in which the complete root is formed, connected to the vascular system of the cutting, and emerges from the cutting (Da Costa et al. 2013; Druege et al. 2019). AR formation in cuttings is controlled by an array of environmental and endogenous factors (Druege et al. 2019; Druege 2020). Plant hormones are key regulators of this developmental process, while primary metabolites such as sugars provide energy and building blocks for the developing roots and, depending on their properties, can also function as signals (Da Costa et al. 2013; Druege et al. 2019; Lakehal and Bellini 2019).

The plant hormone auxin is a central player during AR formation, acting in concert with an array of other plant hormones (reviewed in Da Costa et al. 2013; Druege et al. 2019; Lakehal and Bellini 2019). Auxin, mainly indole-3-acetic acid (IAA), rises in the rooting region during the AR induction phase and acts as a positive regulator of AR induction, whereas high auxin levels inhibit subsequent AR differentiation and growth during the following phases. The wound-responsive hormone jasmonic acid (JA) and its physiologically active conjugate jasmonoyl-isoleucine (JA-Ile) show a transient accumulation in the rooting zone of petunia and pea cuttings shortly after excision (Ahkami et al. 2009; Rasmussen et al. 2015; Jurenic et al. 2026), indicating a positive role of JA during AR induction in cuttings (Druege et al. 2019). At low concentrations, cytokinins have important functions during early cell reprogramming but, at high concentrations, act antagonistically against auxin during AR induction (Da Costa et al. 2013). Based on its function as an inhibitor of cell cycle progression (Wolters and Jürgens 2009), the stress-sensitive hormone abscisic acid (ABA) may inhibit AR induction (Da Costa et al. 2013).

The physiological control of AR formation in hydrangea is underexplored. Our recent study on *H. macrophylla* cuttings focused on the roles of light and carbohydrates in adventitious rooting of two contrasting cultivars (Druege and Chamas 2026). The cultivar ‘Clarissa’, which showed a lower rooting capacity compared to ‘Caipirinha’, revealed lower hexose levels and a lower hexose/sucrose ratio in the 0–1 cm stem base section during the early rooting period (Druege and Chamas 2026). However, the finding that rooting of ‘Clarissa’ could not be rescued by increasing externally supplied hexose doses during the first 24 h after cutting excision points to other endogenous factors limiting AR formation in that cultivar (Druege and Chamas 2026). Comparisons of easy- and difficult-to-root cultivars or species in various plants have highlighted important roles for plant hormones, particularly auxin, in regulating auxin homeostasis and signaling (reviewed in Druege et al. 2019). For example, De Almeida et al. (2015) compared the rooting-recalcitrant species *Eucalyptus globulus* with the easy-to-root species *E. grandis*. *E. globulus* accumulated lower IAA levels in the vascular cambium than *E. grandis*, whereas exogenous IAA restored AR formation in *E. globulus*. Gene-expression analyses revealed differential expression of genes involved in auxin biosynthesis and transport between the two species. In addition, *E. globulus* exhibited higher expression of the auxin-response repressor gene *IAA12*, the corepressor gene *TOPLESS*, and the cytokinin-response regulator gene *ARR1* (De Almeida et al. 2015). These genes have been proposed by the authors to act as negative regulators of adventitious root formation in *E. globulus*. Poor adventitious root formation in a particular carnation cultivar was associated with higher levels of *DcGH3.1* transcripts and IAA–Asp, accompanied by lower IAA levels in the stem base of cuttings during adventitious root induction, compared with a well-rooting cultivar (Cano et al. 2018). The poor-rooting phenotype could be partially rescued by chemically inhibiting GH3 enzyme activity. In a genome-wide association study of rooting performance in 95 rose genotypes, 98, 218, and 4 single-nucleotide polymorphisms (SNPs) were associated with root number, root length, and root biomass, respectively (Nguyen et al. 2020). Some of these SNPs were located in genes homologous to genes known to be involved plant hormone signaling, including *ETHYLENE INSENSITIVE 3-like*, *AUXIN RESPONSE FACTOR 19*, *protein AUXIN RESPONSE 4*, and a *MYC2* transcription factor.

Considering the discussed general importance of plant hormones, the present study followed the hypothesis that the different rooting capacities of the *H. macrophylla* cultivars ‘Clarissa’ and ‘Caipirinha’ may be based on differences in plant hormone regulation, particularly auxin dynamics. Therefore, this study aimed to answer the following questions:

(i) How do the two cultivars respond in terms of rooting to stem base–targeted pulse treatments with IAA, and can rooting of ‘Clarissa’ be rescued by external auxin?
(ii) How does external IAA affect carbohydrate levels and distribution in the stem base?
(iii) What are the early dynamics of the plant hormones IAA, JA, JA-Ile, free base and riboside cytokinins, and ABA in the stem base?

Because the rooting analysis revealed that the length of the rooting zone differed between the two cultivars, carbohydrates and plant hormones were analyzed in two sections of the stem base: the 0–1 cm section and the 1–2.5 cm section below the lowest leaf.

## Material and methods

### Plant material and experiments

Stock plants of *Hydrangea macrophylla* (Thunb.) Ser. ‘Caipirinha’ and ‘Clarissa’ were established from young plants in 2022 and grown in a greenhouse at the Erfurt Research Centre for Horticultural Crops as described by Druege and Chamas (2026). To fulfill a 1000 h × 4 °C chilling requirement during the winter, each year in November, the stock plants were cut back and kept at low temperatures (heating/ventilation at 2 °C/4 °C) under natural photoperiods until March of the following year. Thereafter, the heating/venting temperatures were incrementally raised over two weeks to 20/22 °C during the day and 18/20 °C during the night, which were then maintained throughout the entire growth period. During the growth period, a 16 h photoperiod was provided by high-pressure sodium lamps, while automatic shading by a curtain was applied when outdoor radiation exceeded 30 klx. Depending on the results of substrate analysis, liquid fertilization (0.1–0.3% Hakaphos Soft Spezial 16–8-22(+ 3); Compo, Münster, Germany) was applied up to three times per week. The target N_min_ content in the substrate was 100–150 mg L⁻^1^. Cuttings were harvested at regular intervals during the growth period as described below. In 2023, cuttings from two harvests were used for experiments 1 and 2 to analyze the response of AR formation and carbohydrate homeostasis of the two cultivars to increasing doses of IAA. In 2024, cuttings from one harvest were used for experiment 3 to analyze the dynamics of plant hormones in basal stem sections in relation to AR formation.

### Harvest of cuttings, rooting conditions and auxin treatments

Shoot tip cuttings (softwood cuttings), consisting of a stem bearing one pair of fully developed leaves, younger leaves, and the shoot apex, and with a basal stem (below the lowest leaf) of approximately 2.5 cm in length, were excised from the stock plants, always leaving two leaves or nodes on the stock plant to promote axillary shoot growth. In experiment 2, the length of the basal stem was precisely adjusted to 2.5 cm using a template. Cuttings were immediately planted in trays containing Perligran medium perlite (Knauf Performance Materials GmbH, Dortmund, Germany), and the trays were covered with transparent plastic hoods.

In experiment 1, the cuttings were cultivated in a greenhouse under a 16 h photoperiod provided by high-pressure sodium lamps. Heating and venting temperatures were set to 20 °C and 22 °C, respectively. For shading, the greenhouse glass was externally treated with ReduSol (Redusystems, Baarle-Nassau, The Netherlands). Additionally, inner shading curtains were automatically closed when outdoor radiation exceeded 20 klx, and the trays were covered with a green net. As a result, the average photosynthetic photon flux density (PPFD) at plant level was 27 µmol m⁻^2^ s⁻^1^. In experiments 2 and 3, the cuttings were cultivated in a growth chamber (Johnson Controls, Cork, Ireland) under the following conditions: temperature, 22/20 °C (day/night); humidity outside covered trays, 85/60% (day/night); PPFD, 30 µmol m⁻^2^ s⁻^1^ provided by white fluorescent tubes under a 16 h photoperiod.

For analysis of the AR formation response and carbohydrate homeostasis to increasing doses of IAA, immediately after harvest of the cuttings, their stem bases were immersed in ethanolic IAA solutions (51.7% v/v) up to a height of 2.1–2.2 cm for 1 min and were then planted as described above.

### Assessment of root formation and growth of the basal stem

At 21 days post excision (dpe), the intensity of AR formation was assessed. The percentage of rooted cuttings was calculated by dividing the number of rooted cuttings by the number of planted cuttings. Roots of each cutting were counted and assigned to root length classes (0.5 cm intervals). To calculate root length values, the midpoints of the respective classes were used. For example, roots measuring 2–2.5 cm were assigned a value of 2.25 cm. Average root number and total root length per planted cutting were calculated by dividing the total number of roots and the sum of the class midpoints, respectively, by the number of planted cuttings. Mean root length was calculated by dividing the total root length by the total number of roots. For gravimetric determination of root dry mass, roots were removed from the cuttings, dried for 24 h at 60 °C, and weighed. The length of the stem base below the lowest leaf and the length of the rooting zone, defined as the distance between the emergence site of the lowermost AR, typically located at the cut end, and the emergence site of the uppermost AR, were measured using a ruler. In experiment 2, elongation of the basal stem was calculated as the difference between the precisely adjusted length of 2.5 cm at the time of excision and planting and the length determined at 21 dpe. Each tray represented one biological replicate (*n* = 4, 3, and 3 for experiments 1, 2, and 3, respectively), with each replicate consisting of 10 cuttings in experiments 1 and 2 and 5 cuttings in experiment 3.

### Carbohydrate analysis

In experiment 2, samples for carbohydrate analysis were collected at 7 dpe, 5 h after the onset of the photoperiod. Stem base sections of 0–1 cm and 1–2.5 cm above were excised with a scalpel, weighed, immediately shock-frozen in liquid N₂, and then stored at −80 °C until analysis. For each cultivar and stem base section, eight samples from individual cuttings were collected as biological replicates (*n* = 8). Carbohydrates were analyzed enzymatically using a microplate-based assay modified from Klopotek et al. (2010) according to the protocol of Druege and Chamas (2026).

### UPLC-MS/MS analysis of plant hormones

In experiment 3, stem base sections of 0–1 cm and 1–2.5 cm above were sampled from cuttings of both cultivars at 0 hpe, 0.5 hpe, 8 hpe, 24 hpe, 48 hpe, and 96 hpe, immediately shock-frozen in liquid N₂, and then stored at −80 °C until analysis. Levels of JA, JA-Ile, IAA, and the cytokinins *trans*-zeatin (tZ), *trans*-zeatin riboside (tZR), *cis*-zeatin (cZ), *cis*-zeatin riboside (cZR), isopentenyladenine (IP), isopentenyladenosine (IPR), dihydrozeatin (DHZ), dihydrozeatin riboside (DHZR), as well as ABA were analyzed using ultra-performance liquid chromatography coupled with tandem mass spectrometry (UPLC-MS/MS) following the protocol of Šimura et al. (2018). All used hormone standards were obtained from OlChemIm s.r.o, Olomouc, Czech Republic.

After homogenization of the samples in a vibration mill (Retsch, Haan, Germany), 10 mg of fresh tissue was extracted at 4 °C with 1 mL of extraction solution. The extraction solution consisted of 999.25 µL of 50% aqueous acetonitrile (ACN) and 0.75 µL of an internal standard mixture (IS), containing one stable isotope per each analyzed plant hormone, specified in Supplemental Spreadsheet S1, at a concentration of 50 nmol mL^−1^. This resulted in a final concentration of 37.67 pmol mL^−1^ of each internal standard in the extraction solution. Extraction was performed by shaking at 1200 rpm for 15 min (Thermomixer Comfort, Eppendorf, Hamburg, Germany), followed by 5 min of sonication (VWR Ultrasonic Cleaning Bath, Avantor Sciences, Radnor, PA, USA) and 30 min of rotation (SB2 Rotator, Stuart, Eaton Town, NJ, USA). Extracts were centrifuged (3K30/7, Sigma, Osterode, Germany) at 4 °C and 21,913 g for 10 min. The supernatants were purified by solid-phase extraction (SPE) using Oasis Prime HLB cartridges (1 cm^3^, 30 mg, Waters, Eschborn, Germany). Samples were passed through the cartridges (fraction 1), after which the cartridges were washed with 30% aqueous ACN (fraction 2). Both fractions were combined in 2 mL tubes and concentrated to dryness using a centrifugal evaporator (Refrigerated CentriVap with CentriVap-50 Cold Trap, Labconco, Kansas City, MO, USA). Dried extracts were resuspended in 50 µL of 30% aqueous methanol, vortexed for 30 s, homogenized for 2 min in an ultrasonic bath, and finally centrifuged for 30 min at 4 °C and 21,913 g (Sigma 3K30/7).

Liquid chromatography was performed using a UHPLC-ESI–MS/MS system (Spark Holland, Emmen, The Netherlands) consisting of a binary solvent pump (SPH1299), an autosampler (Alias) with a 15 µL needle, 100 µL sample loop, and 10 µL injection volume (µL pickup injection mode), a column oven (Mistral), and a column configuration comprising an inline stainless-steel filter (0.2 µm pore size), an ACQUITY UPLC CSH C18 VanGuard pre-column (5 mm × 2.1 mm; 1.7 µm particle size), and an ACQUITY UPLC CSH C18 column (150 mm × 2.1 mm; 1.7 µm particle size), both from Waters. Sample vials were maintained at 4 °C. Separation of analytes was achieved using gradient elution with 90% aqueous ACN eluate (A) and 5% aqueous ACN eluate (B) as mobile phases, both containing 0.01% formic acid. The gradient program (flow rate = 0.5 mL min⁻^1^, column temperature = 40 °C) was as follows: 0–1 min, 100% B; 1–2 min, linear gradient to 95% B; 2–5 min, linear gradient to 90% B; 5–13 min, linear gradient to 35% B; 13–17 min, 0% B; 17–20 min, 100% B.

Instrument control was managed using Analyst software (version 1.7.3, AB Sciex, Darmstadt, Germany) with the Symbiosis Pico plugin (version 1.2.0.1, Spark Holland). Detailed gradient conditions and retention times are provided in Supplemental Spreadsheet S1. The ESI–MS/MS analysis was performed on a triple-quadrupole mass spectrometer (QTrap 6500, AB Sciex), which was operated in both positive and negative electrospray ionization modes (ESI+, ESI −). The quantification of the phytohormones was based on multiple-reaction monitoring (MRM) mode. All relevant parameters of the mass spectrometer are listed in Supplemental Spreadsheet S1. Chromatograms were analyzed using MultiQuant software (version 3.0.3 HotFix 4, AB Sciex) based on the internal standard method combined with external calibration. For this purpose, the same IS mix used for the samples was analyzed at the beginning of each batch. The peak areas of the corresponding isotopes were then related to those of external calibration standards of the native hormones using a custom R script in RStudio 2024.12.1+563 “Kousa Dogwood” Release (27771613951643d8987af2b2fb0c752081a3a853, 2025-02-02) for Windows. For each cultivar, stem base section, and time point, five samples from individual cuttings were analyzed as biological replicates (*n* = 5).

### Statistics

Mean values and standard errors for each treatment were calculated using Microsoft Excel (Microsoft, Redmond, WA, USA). Depending on the experimental design, the effects of cultivar, auxin treatment, stem base section, and time were analyzed using two- or three-way ANOVA or Student’s t-test in TIBCO Statistica 13.3 (TIBCO Software Inc., Palo Alto, CA, USA). Statistical significance was assessed at *P* levels of 0.05, 0.01, 0.001, and 0.0001. Significant differences between means were identified using the Newman–Keuls test (*P* < 0.05). The number of replicates is provided with the data. Coefficients of determination (R^2^) for linear regressions were calculated using the trendline function in Microsoft Excel.

## Results

### Auxin response of adventitious root formation

Initially, the hypothesis was tested that the lower rooting capacity of ‘Clarissa’ compared with ‘Caipirinha’ may be due to lower auxin concentrations or reduced auxin signal transduction during the root induction phase. If this were the case, increasing external auxin doses should enhance the endogenous auxin signal intensity during the root induction phase and thus compensate for the reduced rooting capacity of ‘Clarissa’. To test this, ethanolic IAA solutions at different concentrations were applied to the basal stem of cuttings from both cultivars as a 1 min pulse treatment immediately after excision from the donor plants.

Based on experiences with other plant species (Hartmann et al. 2011), in a preliminary experiment, the appropriate range of IAA concentrations was determined using the cultivar ‘Caipirinha’. Therefore, IAA concentrations of 10 mM, 50 mM, and 100 mM were compared with 0 mM, which contained only 51.7% ethanol, and the rooting response was analyzed as described above. Increasing the IAA dose from 10 to 50 mM progressively increased the number, length, and dry mass of formed ARs compared to the 0 mM treatment, whereas the highest dose of 100 mM reduced these parameters compared to the 50 mM treatment (Supplemental Fig. S1). These reductions, together with a slight reduction in rooting percentage and greater variation in root number and total root length compared to all other treatments (Supplemental Fig. S1), indicated that 100 mM IAA was supra-optimal for AR induction.

**Fig. 1:**
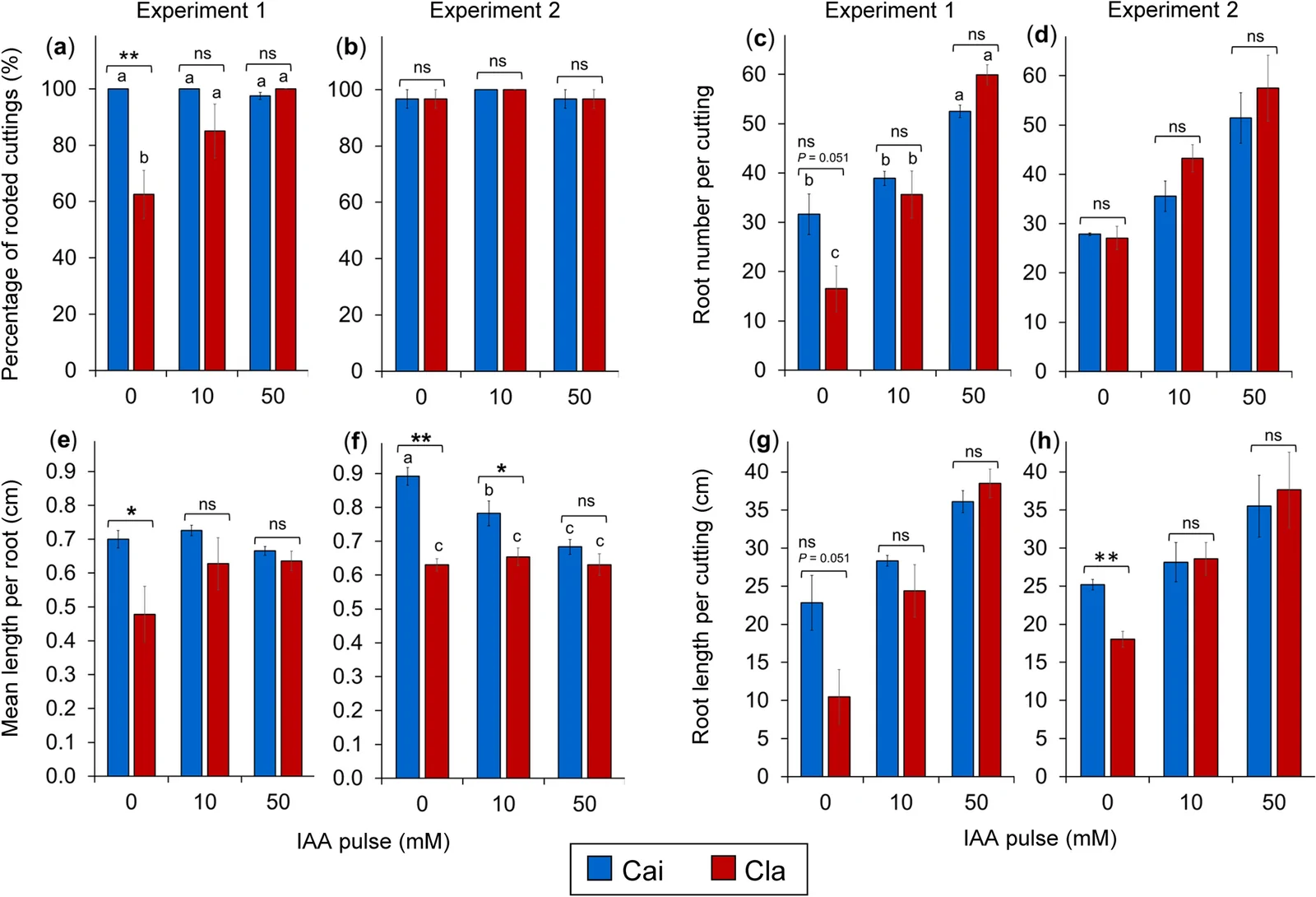
Effect of cultivar and pulsed indole−3-acetic acid (IAA) at different concentrations on the percentage of rooted cuttings **(a, b**), number (**c**,** d**), mean length (**e**, **f**), and total length (**g**,** h**) of adventitious roots of *H. macrophylla*, determined at 21 days post excision. Mean values ± SE. In cases of significant interactions between cultivar and auxin treatment indicated in Supplemental Table S1, different letters mark significant differences across all combinations based on the Newman–Keuls test (*P* < 0.05). One and two asterisks mark significant differences between the two cultivars at the specific IAA concentration at *P* < 0.05 and 0.01, respectively, based on the Student’s t-test (*n* = 4 in experiment 1, *n* = 3 in experiment 2, each *n* consisting of data from 10 cuttings). Cai, ‘Caipirinha’; Cla, ‘Clarissa’; ns, non-significant

**Fig. 2:**
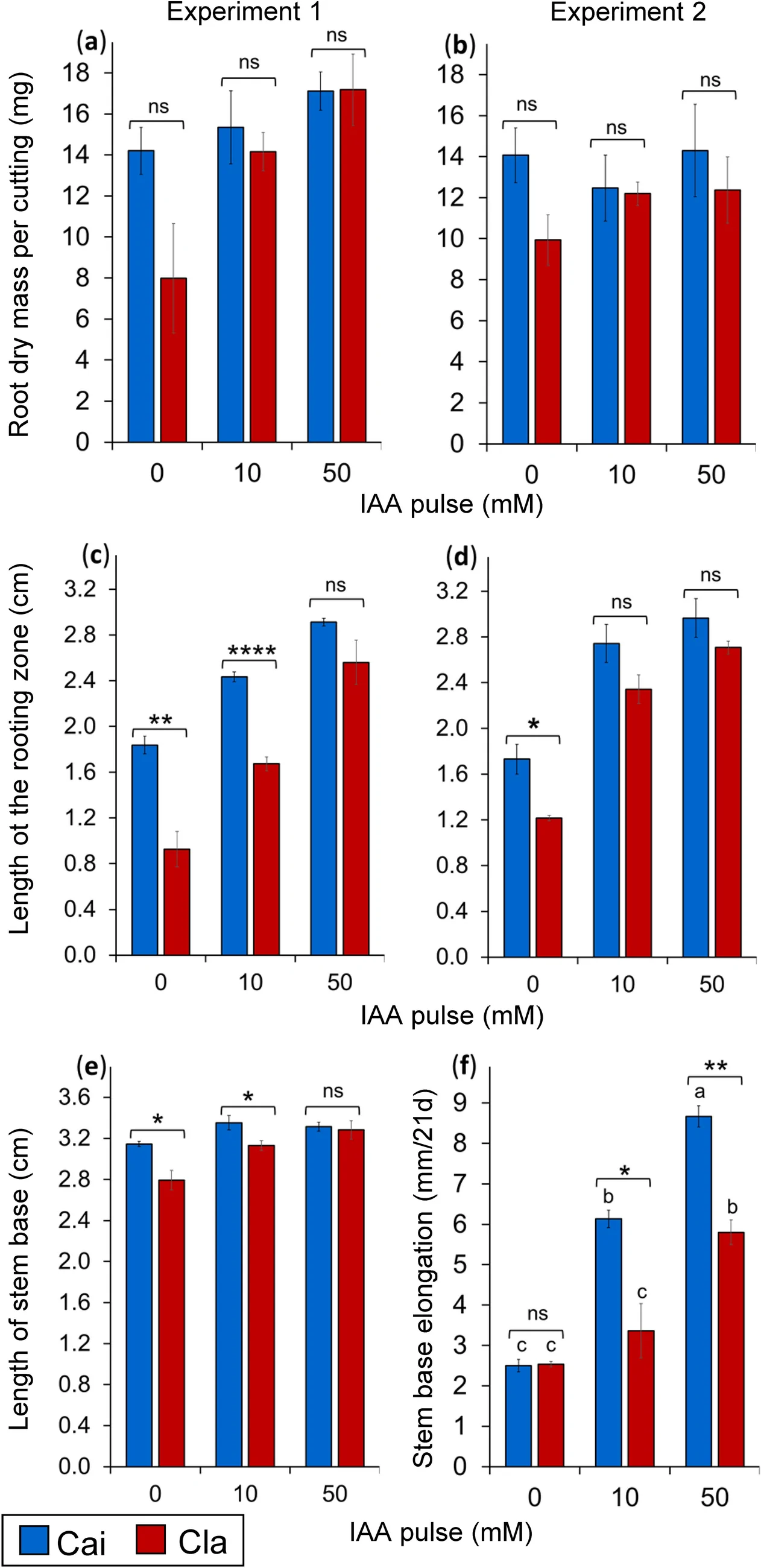
Effect of cultivar and pulsed indole−3-acetic acid (IAA) at different concentrations on root dry mass (**a**, **b**), the length of the rooting zone (**c**, **d**), the length of the stem base (**e**), and stem base elongation (**f**) of cuttings of *H. macrophylla*. Experiments 1 and 2. Determined at 21 days post excision. Mean values ± SE. In cases of significant interactions between cultivar and auxin treatment indicated in Supplemental Table S1, different letters mark significant differences across all combinations based on the Newman–Keuls test (*P* < 0.05). One, two, and four asterisks mark significant differences between the two cultivars at the specific IAA concentration at *P* < 0.05, 0.01, and 0.0001, respectively, based on the Student’s t-test (*n* = 4 in experiment 1, *n* = 3 in experiment 2, each *n* consisting of data from 10 cuttings). Cai, ‘Caipirinha’; Cla, ‘Clarissa’; ns, non-significant

Based on these findings, in experiments 1 and 2, the two cultivars ‘Caipirinha’ and ‘Clarissa’ were exposed to 0 mM IAA, 10 mM IAA, and 50 mM IAA. Further considering that the magnitude of difference in rooting between both cultivars is to some extent sensitive to the environment (Druege and Chamas 2026), the auxin response of rooting of both cultivars was studied in two independent experiments. The rooting data were analyzed at experimental level by two statistical approaches. First, a two-factor ANOVA with the factors cultivar and auxin was applied, and differences between the cultivar–IAA combinations were tested using the Newman–Keuls test. The results of the ANOVA are summarized in Supplemental Table S1. Because the primary interest was the difference between the two cultivars depending on IAA application, the two cultivars were additionally compared at the specified IAA doses using Student’s t-test. The effects of cultivar and IAA application on rooting performance of the two cultivars are illustrated in Fig. 1 and Fig. 2. In experiment 1, without application of IAA, ‘Clarissa’ showed a significantly lower percentage of rooted cuttings (Fig. 1a, according to the Newman–Keuls test and Student’s t-test), a significantly lower number of roots (Fig. 1c, according to the Newman–Keuls test), and a significantly lower mean root length (Fig. 1e, according to Student’s t-test) compared with ‘Caipirinha’. Furthermore, the total length and dry mass of ARs of ‘Clarissa’ reached only about 50% of the levels of ‘Caipirinha’ (Fig. 1g, Fig. 2a). These differences were not statistically significant, while in the case of total root length the *P* < 0.05 threshold of the Student’s t-test was only narrowly missed (Fig. 1g). Increasing doses of IAA enhanced all rooting parameters, except the mean root length in ‘Caipirinha’. However, ‘Clarissa’ benefited more from external IAA. Thus, after application of 10 mM and 50 mM IAA, both cultivars showed the same rooting percentage (Fig. 1a) as well as the same number (Fig. 1c), mean length (Fig. 1e), total length (Fig. 1g), and dry mass (Fig. 2a) of ARs, so that no statistically significant differences were found between the two cultivars.

**Fig. 3:**
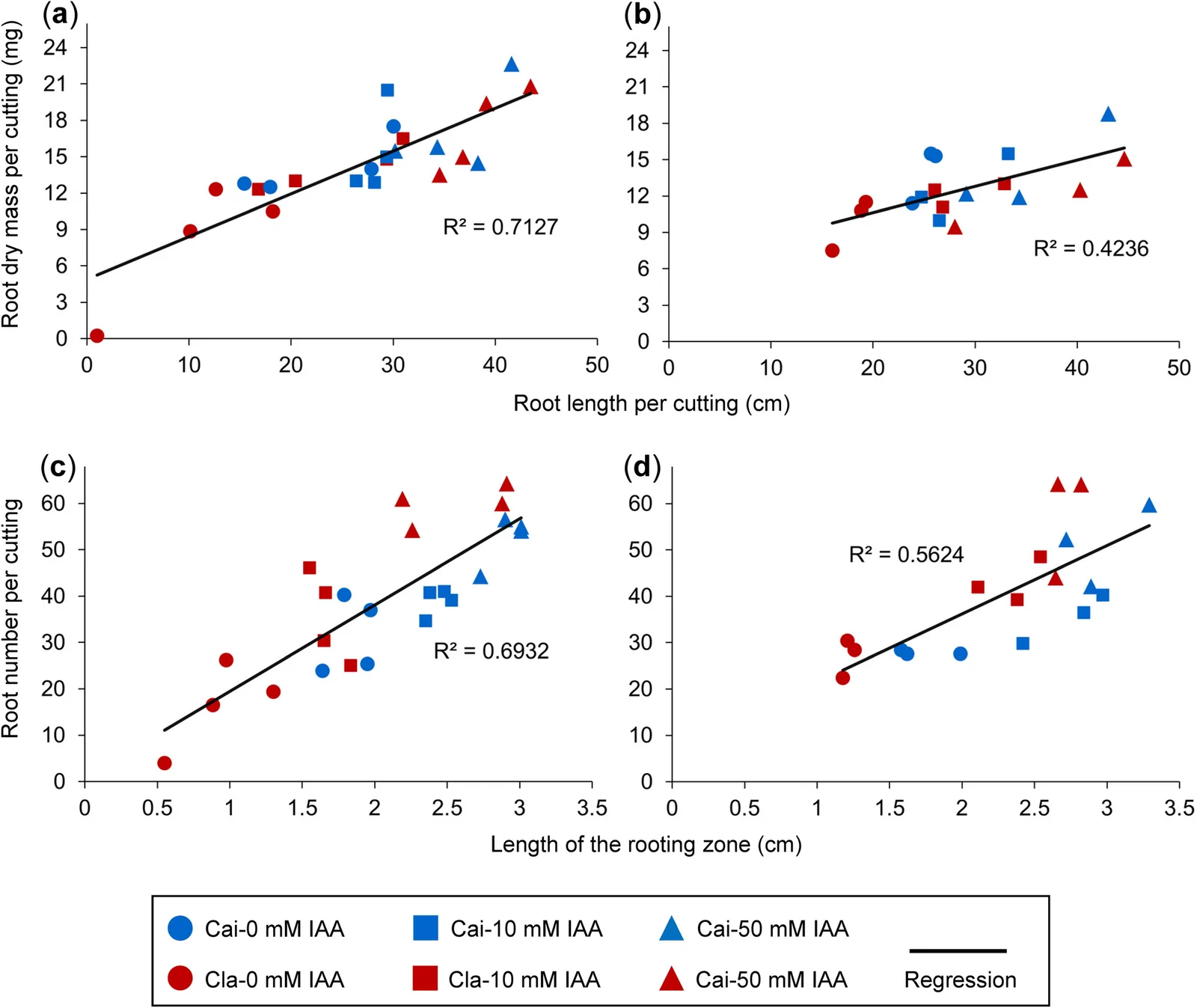
Relationships between root length and root dry mass (**a**, **b**) and between the length of the rooting zone and the number of adventitious roots (**c**, **d**) formed by cuttings of *H. macrophylla* in experiment 1 (**a**, **c**) and experiment 2 (**b**, **d**). Experiment 1, *n* = 24; experiment 2, *n* = 18. Cai, ‘Caipirinha’; Cla, ‘Clarissa’

In experiment 2, rooting was faster compared to experiment 1. Thus, even without IAA application, both cultivars reached almost 100% rooting (Fig. 1b) and produced a similarly high number of roots (Fig. 1d), while ARs were longer compared to experiment 1 (Fig. 1e, f). Nevertheless, in experiment 2 as well, ‘Clarissa’ showed reduced rooting performance in the absence of IAA. Thus, mean length per root (Fig. 1f, according to the Newman–Keuls test and Student’s t-test) and root length per cutting (Fig. 1h, according to Student’s t-test) were significantly lower in ‘Clarissa’ compared to ‘Caipirinha’. Furthermore, the root dry mass of ‘Clarissa’ was only 72% of that of ‘Caipirinha’, even though this difference was not significant (Fig. 2b). Similar to experiment 1, increasing the IAA dose enhanced the number (Fig. 1d), total length (Fig. 1h) and dry mass (Fig. 2b) of ARs on a larger scale in ‘Clarissa’ than in ‘Caipirinha’ so that at 50 mM IAA both cultivars revealed same levels of these parameters. While mean root length remained unaffected by IAA in ‘Clarissa’, this parameter was even reduced by increasing IAA in ‘Caipirinha’ (Fig. 1f). In both experiments, root dry mass was positively correlated with total root length per cutting (Fig. 3a, b). The data points of ‘Clarissa’ were solely distributed on the left side of the regression, when no auxin was supplied, but overlapped with the data points of ‘Caipirinha’ on the right side, when the highest IAA dose was applied. As a whole, both experiments clearly demonstrated that the disadvantage in rooting of ‘Clarissa’ compared with ‘Caipirinha’ is eliminated, when the early auxin signal is enhanced to an adequate level.

Also, the topography of AR formation was analyzed by measuring rooting zone length. In both experiments, when no auxin was applied, the rooting zone of ‘Clarissa’ was significantly shorter than in ‘Caipirinha’, with mean values of 0.93 and 1.22 cm compared to 1.84 and 1.73 cm (Fig. 2c, d, according to Student’s t-test). IAA pulse application increased the length of the rooting zone (Fig. 2c, d). Similar to the other rooting parameters, ‘Clarissa’ responded more strongly to IAA, such that after immersion in 50 mM IAA both cultivars showed a similarly long rooting zone, which exceeded the 2.1–2.2 cm height of the immersion solution, with no significant differences between the cultivars (Fig. 2c, d). In both experiments, the number of ARs formed was positively correlated with the length of the rooting zone (Fig. 3c, d).

The cuttings for experiment 1 were harvested aiming for a basal stem length of 2.5 cm by visual assessment. Measurement of basal stem length at the end of the rooting period revealed a greater length in ‘Caipirinha’ when no auxin was applied (Fig. 2e, according to Student’s t-test). However, auxin application increased the final length of the stem base, indicating that IAA stimulates elongation of the stem base (Fig. 2e, Table S1). To test this, in experiment 2 a basal stem length of 2.5 cm at harvest was ensured by using a template, allowing determination of length increase until the end of the rooting period. This analysis revealed similarly low stem base elongation in the absence of auxin, but an IAA concentration-dependent increase in elongation, which was stronger in ‘Caipirinha’ than in ‘Clarissa’ (Fig. 2f, according to the Newman–Keuls test and Student’s t-test).

### Auxin response of carbohydrate levels and distribution

In an earlier study, it was shown that ‘Clarissa’ showed lower hexose levels and lower hexose/sucrose ratios than ‘Caipirinha’ in the 0–1 cm stem base at 3 days post-excision (dpe) (Druege and Chamas 2026). To obtain information on the carbohydrate status in the stem base at a later stage of rooting and potential auxin–carbohydrate interactions in *H. macrophylla*, the levels of glucose, fructose, sucrose, and starch were analyzed in experiment 2 at 7 dpe. Because the rooting zone of cuttings extended beyond the 0–1 cm section of the basal stem depending on auxin application, and this was particularly evident in ‘Caipirinha’ (Fig. 2c, d), the 0–1 cm section and the upper basal stem section (1–2.5 cm) were analyzed separately.

**Fig. 4:**
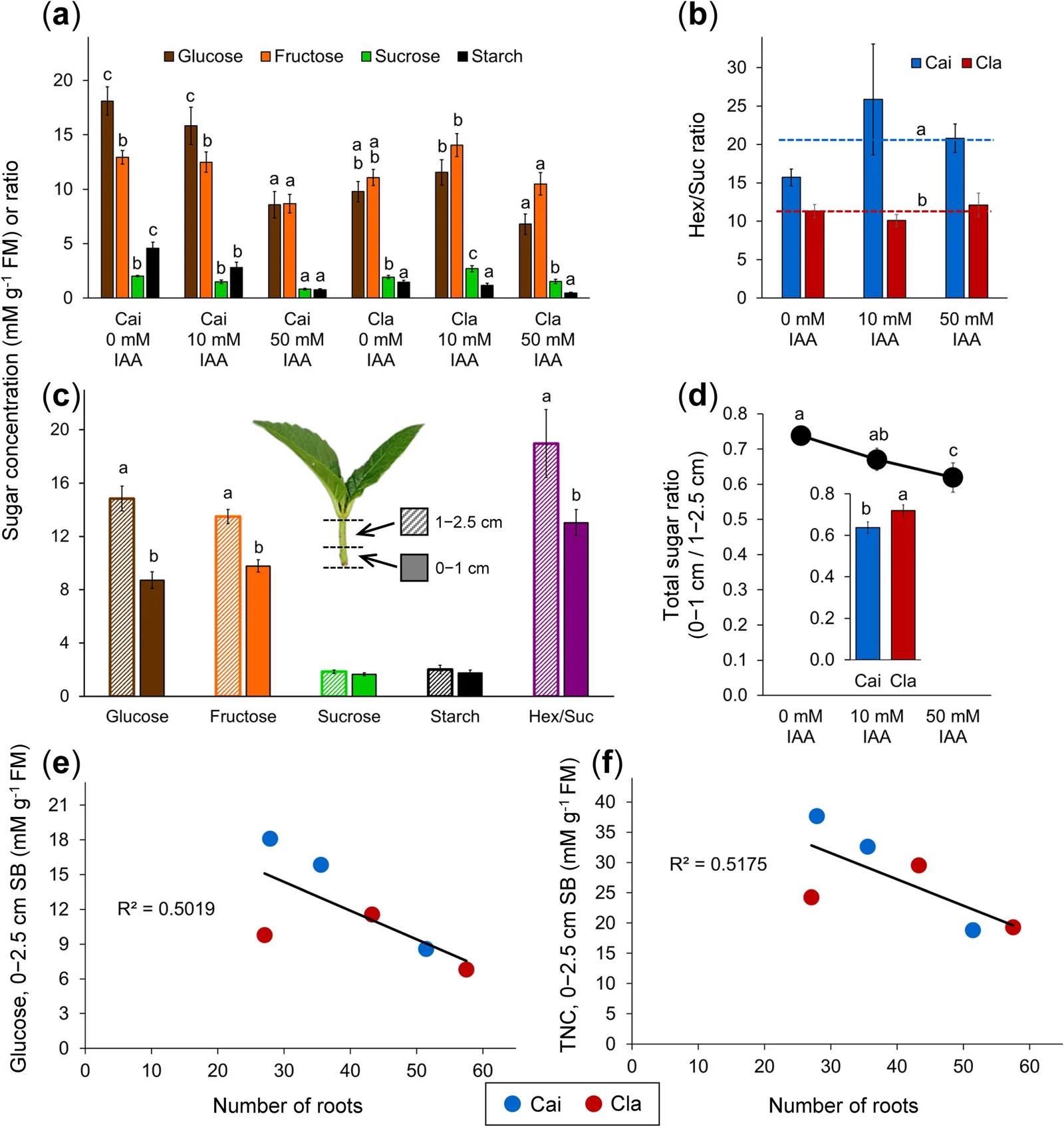
Effects of cultivar and pulsed indole−3-acetic acid (IAA) at different concentrations on carbohydrates in the basal stem sections of cuttings of *H. macrophylla*. Effects of cultivar and IAA pulse on carbohydrate concentrations (**a**) and the ratio of hexose (hex) to sucrose (suc) (**b**) in the 0–2.5 cm basal stem section. Effect of the basal stem section (0–1 cm versus 1–2.5 cm) on carbohydrate levels and the hexose/sucrose ratio (**c**). Effects of IAA pulse and cultivar on the ratio of total sugars between the lower (0–1 cm) and upper (1–2.5 cm) basal stem sections **(d)**. Relationship between the number of roots formed up to 21 dpe and the mean levels of glucose (**e**) and total non-structural carbohydrates (TNC) (**f**) determined at 7 dpe in the 0–2.5 cm basal stem section. Cai, ‘Caipirinha’; Cla, ‘Clarissa’; FM, fresh mass; SB, stem base. **a**–**d** Mean values ± SE. In (**b**), the broken lines illustrate the mean values of Cai (blue) and Cla (red). Different letters indicate significant differences between cultivar–IAA combinations (**a**), between the two cultivars (**b**), between the two basal stem sections (**c**), and between the applied IAA concentrations and cultivars (**d**), based on ANOVA summarized in Supplemental Table S1 and the Newman–Keuls test (*P* < 0.05, *n* = 8). Experiment 2

Independent of the basal stem section, carbohydrate levels were strongly influenced by the interaction between cultivar and auxin application (Supplemental Table S2). When no auxin was applied, ‘Caipirinha’ showed higher glucose and starch levels in the basal stem than ‘Clarissa’ (Fig. 4a). However, pulse treatment with IAA reduced carbohydrate levels, particularly in ‘Caipirinha’, so that after application of 50 mM IAA the stem bases of both cultivars contained the same concentrations of glucose, fructose, and starch, while sucrose was even lower in ‘Caipirinha’ (Fig. 4a). The carbohydrate levels at 7 dpe were negatively correlated with the number of roots formed until 21 dpe, as shown for glucose (Fig. 4e) and total non-structural carbohydrates (Fig. 4f). It is apparent that, similar to the stronger effect of IAA treatment on carbohydrate levels in ‘Caipirinha’ (Fig. 4a), the negative relationships between rooting and carbohydrates were more pronounced in ‘Caipirinha’ than in ‘Clarissa’. Independent of the basal stem section and auxin treatment, the hexose/sucrose ratio was significantly higher in ‘Caipirinha’ compared with ‘Clarissa’ (Fig. 4b, Supplemental Table S2). Comparing the two basal stem sections, independent of cultivar and auxin treatment, lower glucose and fructose levels as well as a lower hexose/sucrose ratio were measured in the 0–1 cm section than in the upper section (1–2.5 cm) (Fig. 4c). Furthermore, auxin affected the distribution of sugars between the two basal stem sections. When IAA was applied, this reduced the ratio of total sugars between the 0–1 cm and 1–2.5 cm stem sections in a concentration-dependent manner (Fig. 4d). In addition, this ratio was higher in ‘Clarissa’ compared with ‘Caipirinha’.

### Dynamic of plant hormone levels and ratios

**Fig. 5:**
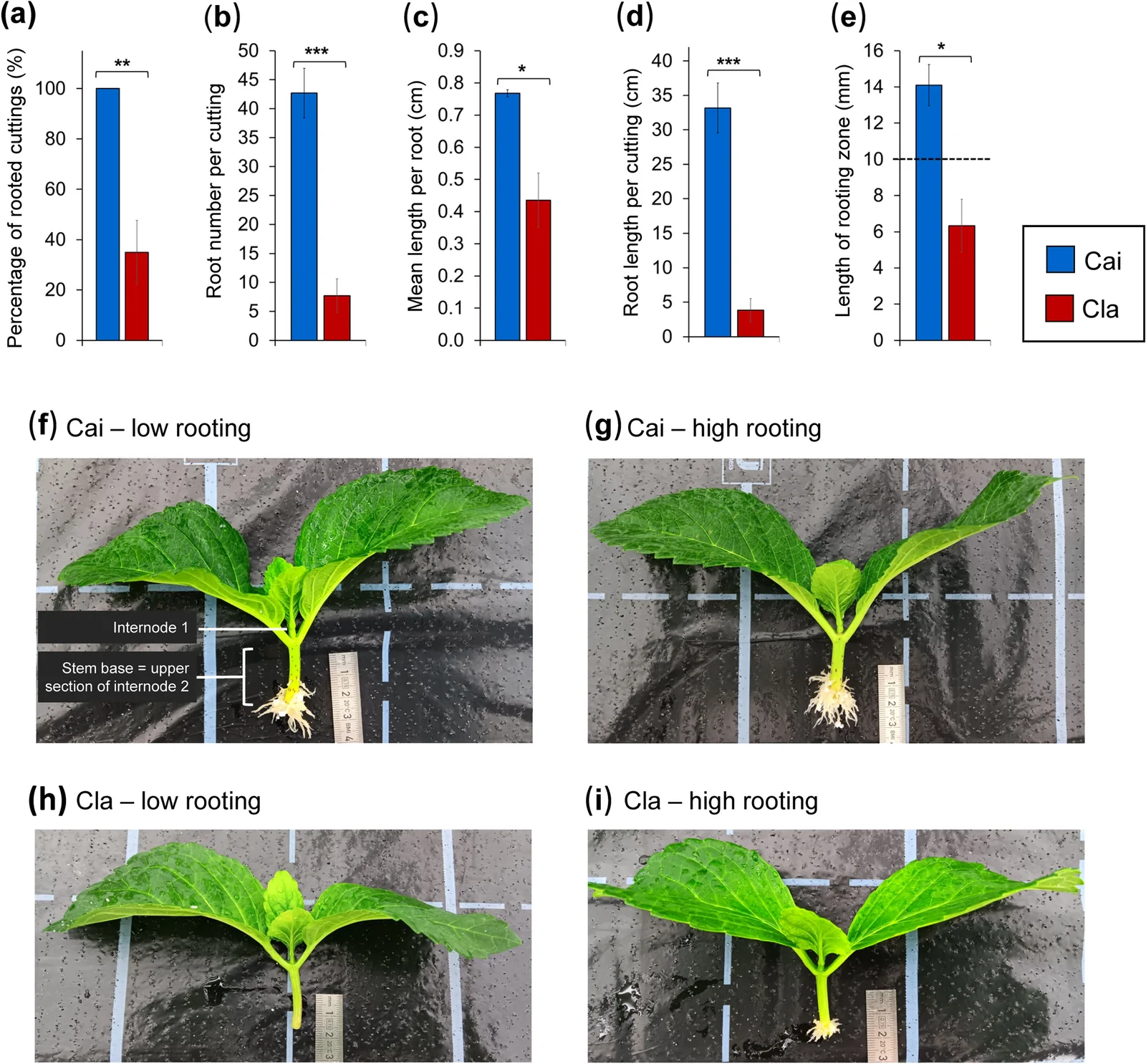
Effects of cultivar on adventitious root formation in experiment 3. Percentage of rooted cuttings (**a**), number (**b**), mean length (**c**), and total length (**d**) of adventitious roots, and length of the rooting zone (**e**) determined at 21 days post excision. Mean values ± SE (*n* = 3, each *n* consisting of 5 cuttings). In (**e**)**,** The broken line indicates the separation of the two basal stem sections for which hormones were analyzed. Photographs of cutting morphology showing representative examples of the lower (**f**) and upper range (**g**) of rooting of ‘Caipirinha’ and of the lower (**h**) and upper (**i**) range of rooting of ‘Clarissa’. In (**f**), the position of the first internode and the upper section of the second internode that corresponds to the stem base are indicated. Cai, ‘Caipirinha’; Cla, ‘Clarissa’

The finding that the rooting deficiency of ‘Clarissa’ compared with ‘Caipirinha’ could be eliminated by a short, early IAA pulse treatment of the stem base suggested either lower endogenous auxin levels or reduced auxin sensitivity in ‘Clarissa’ during root induction. Furthermore, the shorter rooting zone in ‘Clarissa’ compared with ‘Caipirinha’ when no auxin was applied suggested differences in the distribution of the auxin signal or in auxin sensitivity along the basal stem. Given that auxin homeostasis and its activity in AR formation can be influenced by other plant hormones (see Introduction), in the next experiment the most important physiologically active auxin, IAA, JA and its physiologically active conjugate JA-Ile, free-base and riboside-type cytokinins, as well as ABA were analyzed in the 0–1 cm and 1–2.5 cm stem base sections during the 96 h after excision of the cuttings. The final assessment of AR formation in the same experiment confirmed the strong difference between the two cultivars, revealing a significantly higher percentage of rooted cuttings (Fig. 5a), higher number (Fig. 5b), mean length (Fig. 5c) and total length (Fig. 5d) of ARs as well as a longer rooting zone (Fig. 5e) for ‘Caipirinha’ compared to’Clarissa’. Representative photographs in Fig. 5f–g and Fig. 5h–i show the ranges of root morphology of ‘Caipirinha’ and ‘Clarissa’, respectively.

**Fig. 6:**
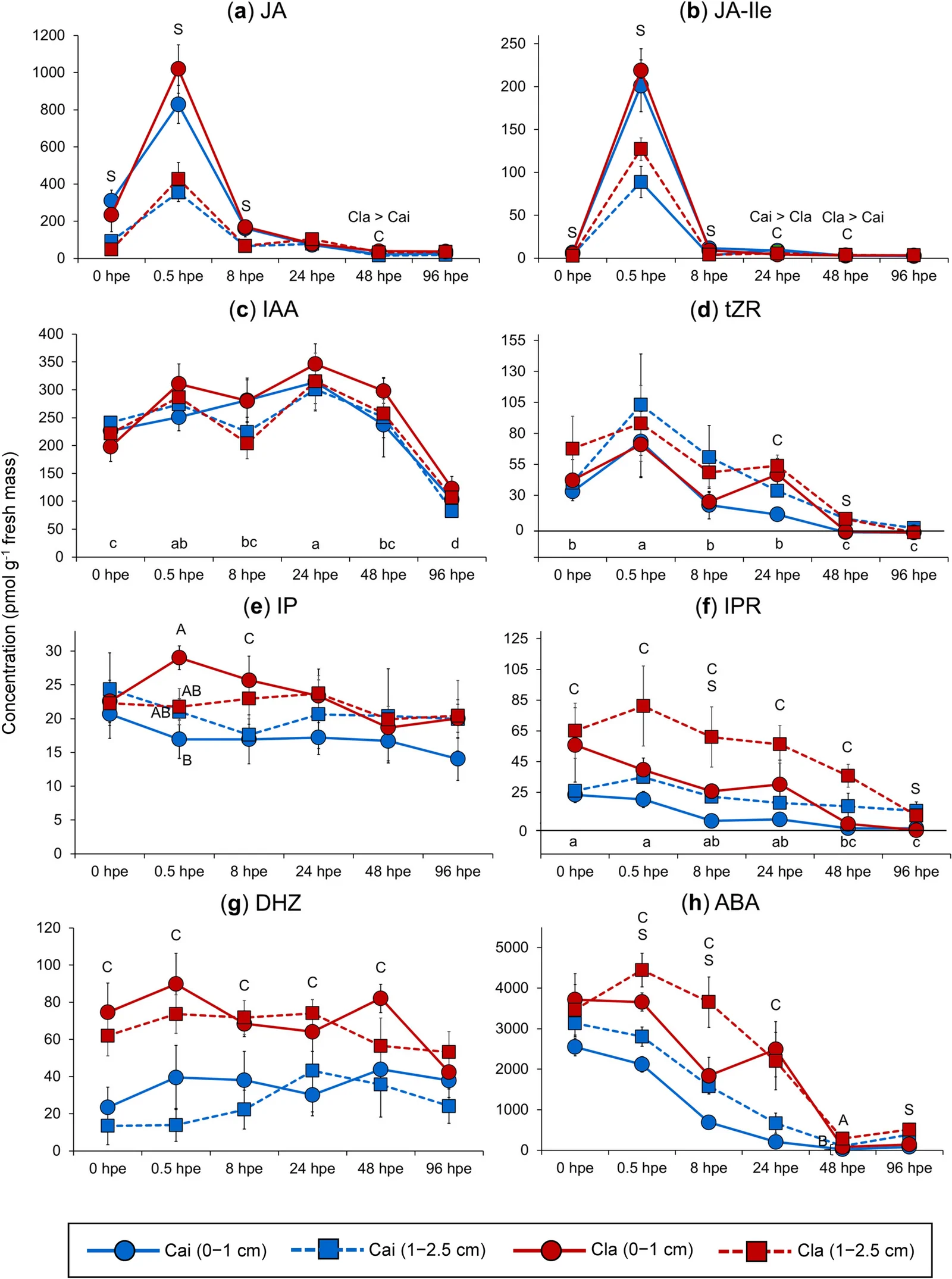
Effects of cultivar and basal stem section on the dynamics of jasmonic acid (JA) (**a**), jasmonoyl-isoleucine (JA-Ile) (**b**), indole-3-acetic acid (IAA) (**c**), *trans*-zeatin riboside (tZR) (**d**), isopentenyladenine (IP) (**e**), isopentenyladenosine (IPR) (**f**)**,** dihydrozeatin (DHZ) (**g**), and abscisic acid (ABA) (**h**) during the first 4 days after excision of *H. macrophylla* cuttings. Mean values ± SE (*n* = 5). In cases of significant effects of time and no interactions between time and other factors, different lowercase letters indicate significant differences between time points based on three-factor ANOVA summarized in Supplemental Table S2 and the Newman–Keuls test (*P* < 0.05). At specified time points, C and S indicate significant effects of cultivar and basal stem section, respectively, while A and B indicate significant differences between specific cultivar–stem section combinations (two-factor ANOVA, Newman–Keuls test, *P* < 0.05). Cai, ‘Caipirinha’; Cla, ‘Clarissa’. Experiment 3

Three-factor ANOVA of the phytohormone data revealed significant effects of cultivar on the levels of tZ, IP, IPR, DHZ, and ABA, of basal stem section on JA, JA-Ile, tZR, and ABA, and of sampling time on JA, JA-Ile, IAA, tZR, IPR, and ABA (Supplemental Table S3). Furthermore, ABA was subject to significant interactions between cultivar and time, while JA and JA-Ile were subject to significant interactions between stem section and sampling time. Figure 6 illustrates the dynamics of the most responsive plant hormones for each cultivar and stem base section. Based on a two-factor ANOVA of cultivar and stem section at each time point, significant effects and differences are indicated. When the factor time was independent of cultivar and stem section, differences between time points are additionally indicated by different letters.

JA showed a strong rise until 0.5 hpe and a subsequent decrease to initial or even lower levels (Fig. 6a). At 0 hpe, 0.5 hpe, and 8 hpe, higher levels were found in the lowermost basal stem section of 0–1 cm compared to the above section. Apart from a slightly higher JA concentration for ‘Clarissa’ compared to ‘Caipirinha’, detected at a very low level at 48 hpe, no cultivar effects were found. A similar trend and almost the same effects were found for JA-Ile, while the levels were much lower than JA (Fig. 6b). Interestingly, no cultivar or stem position effects were found for IAA at any time point (Fig. 6c). However, IAA showed an increase after cutting excision up to the highest levels at 24 hpe and a strong decrease after 48 hpe to the lowest concentrations at 96 hpe (Fig. 6c). Independent of cultivar and stem section, tZR significantly increased after cutting excision until 0.5 hpe and thereafter decreased to the lowest levels measured from 48 hpe onward (Fig. 6d). At 24 hpe, significantly higher tZR levels were found in ‘Clarissa’ compared to ‘Caipirinha’. Isopentenyladenine did not show a clear trend over time (Fig. 6e). However, at 0.5 hpe, the lowermost stem section of ‘Clarissa’ revealed higher IP levels than the respective stem section of ‘Caipirinha’, and at 8 hpe, generally higher IP levels were measured in ‘Clarissa’ independent of the stem section. Isopentenyladenosine decreased over time to significantly lower levels from 48 hpe onward (Fig. 6f). Between 0 and 48 hpe, independent of the stem section, significantly higher IPR levels were measured in ‘Clarissa’ compared to ‘Caipirinha’. At 8 hpe and 96 hpe, higher IPR levels were measured in the upper basal stem section compared to the 0–1 cm section. Similar to IP, the concentration of DHZ remained at a similar level over time (Supplemental Table S3, Fig. 6g). However, similar to IPR, DHZ showed a strong cultivar effect (Supplemental Table S3). Between 0 and 48 hpe, independent of the stem base section, higher DHZ levels were measured in ‘Clarissa’ than in ‘Caipirinha’ (Fig. 6g). The free base tZ as well as cZR and DHZR were not detected or were present at extremely low levels without showing any effect of cultivar or stem section at any time point (Supplemental Fig. S2). *Cis*-zeatin could not be detected in any sample (Supplemental Table S3). The concentrations of ABA were highest among the measured plant hormones at the time of cutting excision but thereafter showed a decrease to low levels until 48 hpe, while this decrease was much faster in ‘Caipirinha’ than in ‘Clarissa’ (Fig. 6h). As a result, independent of the stem base section, higher ABA levels were detected in ‘Clarissa’ at 0.5 hpe, 8 hpe, and 24 hpe. At 0.5 hpe and 8 hpe, independent of cultivar, higher ABA levels were measured in the upper stem sections compared to the 0–1 cm section. At 48 hpe, the upper stem section of ‘Clarissa’ contained higher ABA levels than the 0–1 cm section of the same cultivar and both stem sections of ‘Caipirinha’ (Fig. 6h).

Intercorrelations were analyzed between the individual hormones that were detected throughout the samples (Supplemental Table S4). JA and JA-Ile showed the highest intercorrelation of 0.932 and were also positively correlated with tZR at a moderate level, with coefficients of 0.425 and 0.434. ABA levels were positively correlated with JA and JA-Ile at a moderate level (coefficients of 0.437 and 0.368) and at a relatively high level with the cytokinins tZR and IPR (coefficients of 0.589 and 0.610). IAA showed only weak correlations with the other hormones. Furthermore, intercorrelations at a moderate level were found between certain cytokinins.

**Fig. 7:**
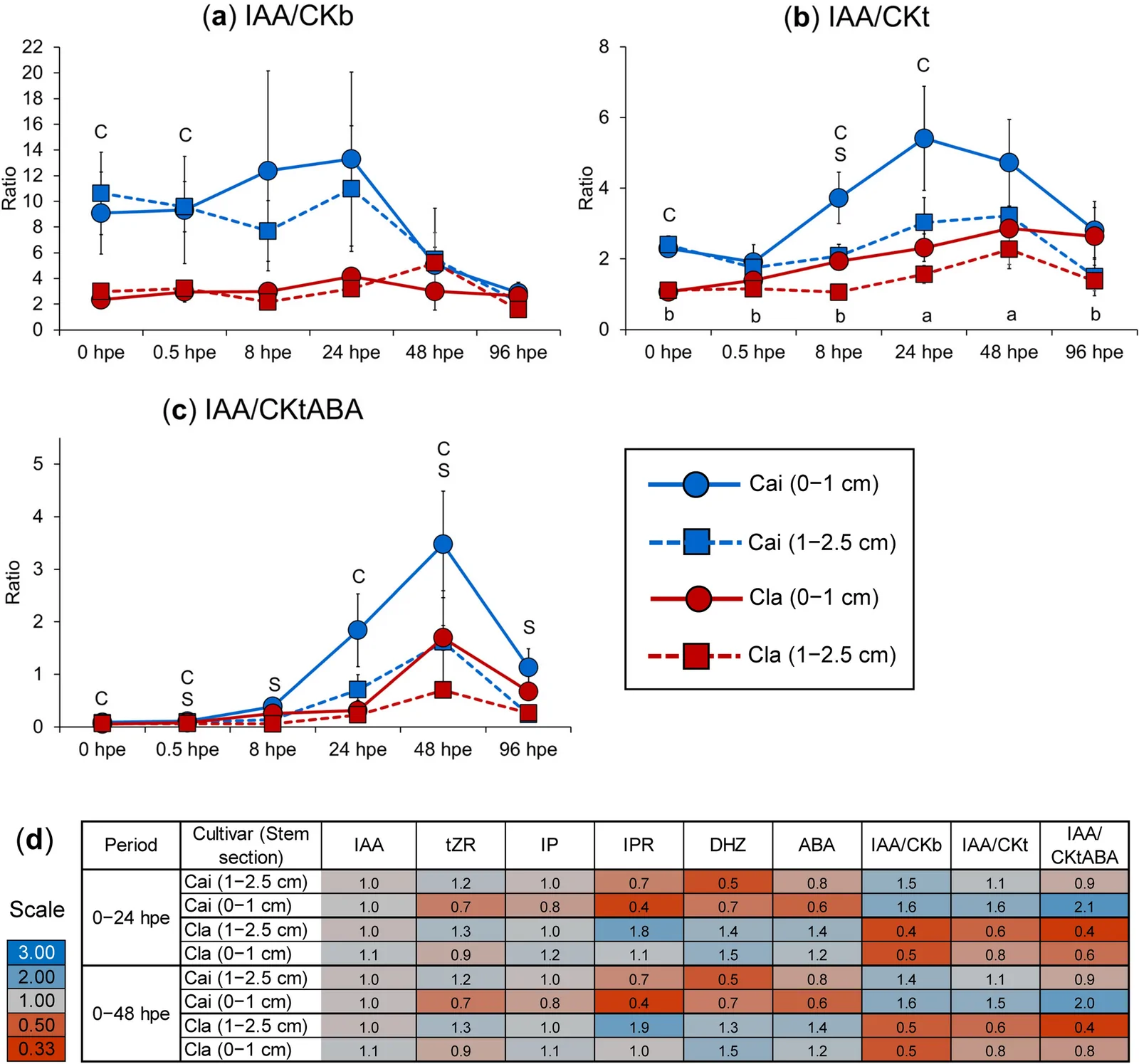
Effects of cultivar and basal stem section on phytohormone ratios. Ratio of indole-3-acetic acid (IAA) to the sum of the cytokinin bases (CKb) *trans*-zeatin, isopentenyladenine, and dihydrozeatin (IAA/CKb) (**a**), ratio of IAA to total CKs (CKt), defined as the sum of CKb plus *cis*-zeatin riboside, *trans*-zeatin riboside, and dihydrozeatin riboside (IAA/CKt) (**b**), ratio of IAA to CKt plus abscisic acid (IAA/CKtABA) (**c**). Heatmap of temporal means calculated over 0–24 h post excision (hpe) and 0–48 hpe for specific hormones and hormone ratios as affected by cultivar and basal stem position (**d**). In (**a**-**c**), mean values ± SE (*n* = 5) are presented, while different lowercase letters indicate significant differences between time points based on three-factor ANOVA summarized in Supplemental Table S2 and the Newman–Keuls test (*P* < 0.05). At specified time points, C and S indicate significant effects of cultivar and stem section, respectively (two-factor ANOVA, *P* < 0.05). In (**d**), normalized values relative to the mean of the four combinations of cultivar and stem base positions are presented (*n* = 5). Cai, ‘Caipirinha’; Cla, ‘Clarissa’. Experiment 3

Regarding the found promotive influence of early IAA application on AR formation of *H. macrophylla*, particularly in the case of ‘Clarissa’ (Fig. 1), and the well-known function of IAA as an AR inducer on the one hand, and considering the possible antagonistic inhibitory functions of cytokinins and ABA during AR induction on the other hand (see the Introduction), the ratios of IAA to free cytokinin bases (IAA/CKb), to total cytokinins as the sum of all detected free cytokinin bases and ribosides (IAA/CKt), and to the sum of total cytokinins plus ABA (IAA/CKtABA) were calculated depending on cultivar, stem section, and time (Supplemental Table S3). All ratios were highly dependent on cultivar. The IAA/CKt and IAA/CKtABA ratios were also highly dependent on stem section and time, while the latter was further subject to an interaction between cultivar and time. The dynamics of these ratios depending on cultivar and stem section are illustrated in Fig. 7a–c. Independent of the stem section, IAA/CKb was higher in ‘Caipirinha’ between 0 and 24 hpe, with a significant cultivar effect at 0 and 0.5 hpe (Fig. 7a). After inclusion of cytokinin ribosides, the resulting IAA/CKt ratios showed an increase after 0.5 hpe, peaking between 24 and 48 hpe, and a decrease thereafter (Fig. 7b). Significantly higher IAA/CKt ratios were found for ‘Caipirinha’ at 0 hpe, 8 hpe, and 24 hpe and in the lowermost stem sections compared to the upper sections at 8 hpe. Including ABA in the ratio further sharpened the dynamics. The IAA/CKtABA ratios increased after excision to peak at 48 hpe and decreased to low levels thereafter, while strong effects of cultivar and stem section became apparent (Fig. 7c). Thus, independently of the stem base section, ‘Caipirinha’ showed a higher IAA/CKtABA ratio than ‘Clarissa’ at 0 hpe, 0.5 hpe, 24 hpe, and 48 hpe. Independently of cultivar, the lowermost stem sections showed a higher IAA/CKtABA ratio at 0.5 hpe, 8 hpe, 48 hpe, and 96 hpe than the upper sections. The correlation matrix in Supplemental Table S4 indicates that the IAA/CKb ratio was primarily dependent on IAA, IP, and DHZ, and the IAA / CKt ratio was additionally affected by IPR. ABA had a predominant effect on the IAA/CKtABA ratio, while correlations with IAA and the above-mentioned cytokinins were still significant.

To integrate the hormonal data over time, we calculated the temporal means of the hormone levels and ratios over the periods 0–24 hpe and 0–48 hpe. The temporal means per combination of cultivar and stem section were further normalized per time period by calculating the ratio of each combination to the respective mean of the four combinations. The results are illustrated in a heat map (Fig. 7d), while the corresponding rooting data of the same experiment are shown in Fig. 5a–e. It becomes apparent that the normalized temporal means of IAA alone did not show any contrast between the two cultivars or stem sections (Fig. 7d). The normalized temporal means of cytokinins and ABA showed a strong contrast between the two cultivars, with lower cytokinin and ABA values for the better-rooting ‘Caipirinha’ (Fig. 7d). However, these values showed only small variation between the two stem sections, even though ARs were mostly, and in the case of ‘Clarissa’ exclusively, formed at the 0–1 cm section (Fig. 5e). By contrast, the normalized temporal means of the IAA-to-antagonist ratios responded to both cultivar and stem section. Among these, the IAA/CKtABA ratios calculated over the two time periods (Fig. 7d) best corresponded to the different rooting capacity and the different topography of AR formation between the two cultivars (Fig. 5). The highest IAA/CKtABA ratios, attained in the basal 0−1 cm stem section of ‘Clarissa’, compared to much lower ratios in ‘Caipirinha’, resulted in intensive AR induction, leading to higher root numbers than in ‘Clarissa’. Whereas the much lower IAA/CKtABA ratio in the above 1−2.5 cm stem section of ‘Clarissa’ (Fig. 7d) corresponded to the failure of AR formation above 1 cm (Fig. 5f), the higher ratio in the respective stem section of ‘Caipirinha’ reached the same level as in the 0−1 cm stem section of ‘Clarissa’ and corresponded to AR emergence also above 1 cm.

## Discussion

### The contrast in rooting of ‘Caipirinha’ and ‘Clarissa’ depends on the intensity of the early auxin signal, which also controls the elongation of the stem base

In a previous study that focused on carbohydrates, the contrast in rooting between the two *H. macrophylla* cultivars ‘Caipirinha’ and ‘Clarissa’ could not be compensated by external sugars (Druege and Chamas 2026). Therefore, in the present study, the potential role of plant hormones in the rooting competence of the two cultivars was investigated. Considering the generally accepted important role of auxin as an inducer of ARs (reviewed in Da Costa et al. 2013; Lakehal and Bellini 2019; Druege et al. 2019), we first investigated whether the contrast in rooting between the two cultivars depends on the intensity of the early auxin signal, which was modified by short immersion of the stem bases of cuttings in ethanolic solutions of IAA at different concentrations and by analyzing the rooting response. The findings that both cultivars showed an increase in root number (Fig. 1c, d) and root length per cutting (Fig. 1g, h) with increasing IAA doses up to 50 mM document the important role of the early auxin signal in AR formation of *H. macrophylla*. Applying IAA at defined concentrations during the early rooting period clearly triggered AR induction and thus produced clearer responses than the small and variable rooting response of an unspecified *Hydrangea* line to increasing amounts of IAA supplied to different rooting media up to 400 µmol kg^−1^ substrate (Szajdak et al. 2015). However, similar to the present results, spraying cuttings of two cultivars of *H. paniculata* with an aqueous solution of 200 mg L^−1^ IBA or inserting the cutting bases in 1% IBA powder enhanced the percentage of rooted cuttings and the degree of rooting (Pacholczak and Nowakowska 2017). The observed weak, absent, or, in the case of ‘Caipirinha’, even negative response of mean root length to increasing IAA dose (Fig. 1e, f) is consistent with the current understanding that IAA concentrations effective for induction are rather inhibitory to AR elongation (Da Costa et al. 2013). Obviously, IAA application, even though limited to 1 min after excision, affected not only induction but also the subsequent growth of the developing roots in ‘Caipirinha’.

The finding that, in the absence of auxin application, ‘Clarissa’ exhibited less rooting than ‘Caipirinha’ at 21 dpe in three independent experiments − one conducted in a greenhouse and two in a growth chamber − confirmed the lower rooting capacity of ‘Clarissa’ (Druege and Chamas 2026). In experiment 2, however, a statistical difference between the cultivars was found only in root length, while both cultivars achieved a rooting percentage of almost 100%. This result indicates a generally faster root development in that experiment. Because the donor plants were grown in a greenhouse, variable environmental factors at the stock plant level, such as light (Klopotek et al. 2012), and also plant age (Rasmussen and Hunt 2013; Rasmussen et al. 2015) may have affected AR formation. Nevertheless, the significantly lower root length and substantially lower root dry mass recorded for ‘Clarissa’ at 21 dpe indicate delayed root development relative to ‘Caipirinha’ in experiment 2 as well.

The finding that in two independent experiments rooting of both cultivars was brought to the same level by 1 min immersion of the stem bases in 50 mM IAA (Fig. 1, Fig. 2a, b), clearly documents that the rooting deficiency of ‘Clarissa’ is related to auxin. However, the compensation of the rooting deficiency by the externally stimulated increase in early auxin signal intensity could be based on either supplementation of lower endogenous auxin levels or a lower sensitivity to auxin that requires a stronger auxin signal. Furthermore, the shorter rooting zone of ‘Clarissa’ versus ‘Caipirinha’, which was also compensated by external IAA (Fig. 2c, d) and strongly influenced the total number of roots in an auxin-dependent manner (Fig. 3c, d), suggests a different distribution of the auxin signal or signal transduction along the stem base between the two cultivars.

Stem elongation is determined by early cellular activity in the shoot apical meristem and subsequent cell expansion in the internodes, which depends in part on cell-wall loosening (Chen et al. 2026). The similar increase in the length of the stem base, even in the absence of auxin during the rooting period (Fig. 2f), indicates that elongation growth remained active in this part of the cutting. This growth likely reflects the relatively young developmental stage of the stem base, which corresponds to the upper section of the second internode of the young apical shoot (Fig. 5f). The finding that IAA application stimulated the elongation of the stem base of both cultivars (Fig. 2f) provides evidence for a novel role of auxin during AR formation in cuttings and is consistent with the well-known stimulatory function of auxin in stem elongation (Cleland 2010; Chen et al. 2026).

### Carbohydrate utilization in the stem base of *H. macrophylla* depends on the intensity of AR formation, which is enhanced by auxin

Our finding that, independent of the stem section, ‘Clarissa’ revealed lower glucose and starch levels than ‘Caipirinha’ at 7 dpe in the absence of auxin application (Fig. 4a) confirms earlier findings in the 0−1 cm stem base section at 3 dpe (Druege and Chamas 2026) and indicates lower sucrose utilization and hexose availability compared to ‘Caipirinha’ in both basal stem regions at a later stage of AR formation. Depending on the balance between auxin-stimulated carbohydrate influx into the stem base, resulting from enhanced sink activity through activation of sucrolytic enzymes (Ahkami et al. 2013; Agulló-Antón et al. 2014), and the downstream utilization of carbohydrates during auxin-stimulated AR development, auxin application to cuttings can enhance or decrease carbohydrate concentrations in the stem base, as shown for carnation and petunia, respectively (Agulló-Antón et al. 2011; Ahkami et al. 2013). Our finding that increasing the IAA dose up to 50 mM reduced glucose and starch concentrations in the stem base, particularly in ‘Caipirinha’ (Fig. 4a), while rooting was correspondingly promoted in both cultivars (Fig. 1d, h), and the negative relationship between AR number and carbohydrate levels (Fig. 4e, f), strongly suggests that auxin reduced carbohydrate levels in *H. macrophylla* at 7 dpe by stimulating carbohydrate utilization driven by auxin-promoted AR development. This conclusion is supported by a study by Zhang et al. (2017) on IAA-dependent carbohydrate dynamics in the basal (2 cm) phloem tissue of apple cuttings. Immersion of the 2 cm bases of cuttings of *Malus hupehensis* in 100 mg L^−1^ IAA for 2 h prior to planting significantly enhanced adventitious rooting, as determined at 72 dpe (Zhang et al. 2017). This was associated with higher consumption of sugars and starch, so that after a transient IAA-induced increase in sugar concentrations, these decreased to minimum levels that were slightly lower and were reached 18 days earlier than in the control cuttings (Zhang et al. 2017). Our finding that the auxin-induced decrease in carbohydrate levels and the negative relationship between root number and carbohydrate levels were less pronounced in ‘Clarissa’ than in ‘Caipirinha’ (Fig. 4a, e, f), even though auxin stimulation of rooting was stronger than in ‘Caipirinha’ (Fig. 2h), indicates that AR formation in ‘Clarissa’ is less dependent on carbohydrates than in ‘Caipirinha’. This corresponds to the earlier finding that rooting of ‘Caipirinha’ benefited from external hexose, whereas ‘Clarissa’ did not (Druege and Chamas 2026).

The lower hexose levels and lower hexose/sucrose ratio found in the lowermost 0–1 cm basal stem section compared to the 1–2.5 cm section (Fig. 4c) indicate more intensive carbohydrate utilization in the basal 0–1 cm section than in the section above. This is probably based on more intensive AR formation in the lowermost part of the stem base, which, in the case of ‘Clarissa’, was almost the exclusive region of root emergence when no auxin was applied (Fig. 2d). However, wound healing that is also regulated by auxin (Lup et al. 2016) may have additionally enhanced carbohydrate consumption in the 0–1 cm section. According to this view, wounding of carrot tissue increased respiration, caused losses of glucose and fructose, and activated different metabolic pathways, including the TCA cycle (Han et al. 2017). The finding that increasing external auxin supply reduced the sugar ratio between the lowermost 0–1 cm and the above basal stem section (Fig. 4d) indicates that the applied auxin stimulated carbohydrate utilization particularly near the cut surface of the stem. Because IAA was applied by a 1-min immersion of the stem base up to a height of 2.1–2.2 cm, it can be expected that most IAA entered the stem base via the cut surface, such that more IAA likely reached the 0–1 cm section than the section above.

Overall, the carbohydrate data in the context of the rooting results indicate intensive carbohydrate utilization in the stem base up to 7 dpe, which is more pronounced near the cut surface and is evidently the result of cultivar-dependent and auxin-driven AR formation.

### Not endogenous IAA, but cytokinins and ABA, make the difference between ‘Caipirinha’ and ‘Clarissa’, probably acting as IAA antagonists during AR induction

The early rise in JA and its physiologically active, conjugated form JA-Ile, peaking at 0.5 hpe and rapidly decreasing thereafter (Fig. 6a, b), is consistent with similar dynamics observed in cuttings of *Petunia hybrida* ‘Mitchell’ (Ahkami et al. 2009), two parental petunia species (Jurenic et al. 2026), and pea (Rasmussen et al. 2015). This is likely based on the well-known stimulation of JA biosynthesis in response to wounding (Wasternack and Hause 2013). However, the similar dynamics and peak levels of JA and JA-Ile in both cultivars, together with the very high correlations between JA and JA-Ile across both cultivars (Supplemental Table S4), do not indicate that JA biosynthesis or its conversion to the physiologically active form contributes to the different rooting capacity of the two cultivars. Surprisingly, IAA, as the most important physiologically active auxin, did not differ between cultivars or stem sections (Supplemental Table S3, Fig. 6c). However, considering the important function of auxin as an AR inducer and its known inhibitory role in subsequent AR differentiation and growth (Da Costa et al. 2013), which was also apparent in the present study (Fig. 1f), the significant increase in IAA after excision to a peak at 24 hpe and the marked decrease between 48 and 96 hpe suggest that the induction phase in both cultivars occurred before 96 hpe.

Considering that auxin does not act independently during AR induction but in cooperation with other hormones, among which cytokinins play an outstanding role (Da Costa et al. 2013; Lakehal and Bellini 2019), we analyzed important cytokinin free bases and ribosides. The classical view is that after excision of cuttings, cytokinin levels should decrease in the stem base because cytokinin delivery from the root system is interrupted (Steffens and Rasmussen 2016). According to this theory, the levels of tZR and IPR decreased to significantly lower levels from 0.5 hpe onward (Fig. 6d, f). However, cytokinins are not only synthesized in roots but also in aerial tissues such as leaves, stems, and flowers, where tZR is transported acropetally in xylem sap, while IPR is transported in the phloem and can move in both basipetal and acropetal directions (Kudo et al. 2010; Skalicky et al. 2018). The earlier transient increase in tZR up to 0.5 hpe (Fig. 6d) may indicate a wound response. This view is supported by findings in Arabidopsis, where mechanical wounding of hypocotyls stimulated cytokinin biosynthesis and increased cytokinin levels, including tZR (Ikeuchi et al. 2017).

Among the free bases, which are biologically active (Sakakibara 2010), IP and DHZ were present at significant concentrations and remained relatively constant over time (Fig. 6e, g). According to current understanding, cytokinins can stimulate the cell cycle at very early stages and, depending on the plant, microcallus formation can be involved, but they act antagonistically to auxin during AR induction, during which reprogramming occurs leading to the specification of root founder cells (Da Costa et al. 2013; Bustillo-Avendaño et al. 2018; Druege et al. 2019; Lakehal and Bellini 2019). The finding that ‘Clarissa’ exhibited higher cytokinin levels than ‘Caipirinha’ in the basal stem during the period from 0 to 48 hpe suggests that higher cytokinin levels antagonized IAA during AR induction. This particularly applied to DHZ and IPR, but also to tZR at 24 hpe and to IP at 8 hpe and at 0.5 hpe in the lowermost stem section, thereby inhibiting AR formation compared with ‘Caipirinha’. Additionally, the lower IPR levels in the lowermost basal stem section compared with the 1–2.5 cm stem section (Supplemental Table S3, Fig. 6f) may have contributed to the observation that rooting of the cuttings was predominantly distributed along 0–1 cm, particularly in the case of ‘Clarissa’ (Fig. 5e).

Following the classical view that only the free bases of cytokinins are biologically active, there is increasing evidence that cytokinin ribosides are also biologically active (Nguyen et al. 2021), although this view is still under discussion (Romanov and Schmülling 2022). Considering both possibilities, the ratios of IAA to the free bases (IAA/CKb) and to total cytokinins including the ribosides (IAA/CKt) were calculated. The IAA/CKb ratios were higher for ‘Caipirinha’ between 0 and 24 hpe but were not affected by the stem base section (Fig. 7a). This could explain the better rooting of ‘Caipirinha’ (Fig. 5a–d) but not the preferential root emergence from the lowermost stem section (Fig. 5e). However, including ribosides in the ratio (Fig. 7b) revealed a peak pattern, with the highest IAA/CKt ratios between 24 and 48 hpe. The higher ratios observed for ‘Caipirinha’ and for the lowermost stem section could explain the better rooting of ‘Caipirinha’ and, to some extent, also the preferential root emergence from the basal 0–1 cm. These findings, together with the observed IAA response of rooting (Fig. 1), strongly support an important role of cytokinins in the regulation of AR formation in *H. macrophylla* by antagonizing the AR inducer IAA during AR induction.

Interestingly, a recent study by Mao et al. (2023) shed new light on a mechanism of cytokinin–auxin antagonism during AR induction in apple that is located downstream of the auxin signal. First, analysis of auxin and cytokinins revealed that better rooting of microcuttings from easy-to-root apple rootstocks compared to difficult-to-root genotypes was associated with lower levels of ZR and higher IAA/ZR ratios at 2 dpe. Furthermore, AR formation was negatively related to the cytokinin-stimulated expression of the *TEOSINTE BRANCHED1*, *CYCLOIDEA*, and *PCF* gene *MdTCP17*, whose overexpression inhibited AR formation (Mao et al. 2023). MdTCP17 acted by reducing the binding of the auxin-responsive transcription factor MdWOX11 to the promoter of the auxin-responsive lateral organ boundaries domain gene *MdLBD29*, whose expression was positively correlated with AR formation (Mao et al. 2023).

Regarding the inhibitory role of ABA in cell cycle progression (Wolters and Jürgens 2009), the higher ABA concentrations in ‘Clarissa’ and in the upper basal stem section of 1–2.5 cm compared to the lowermost section (Fig. 6h) support the conclusion that higher ABA levels exert an additional inhibitory influence on AR formation in ‘Clarissa’ and in the upper basal stem. This is further supported by the IAA/CKtABA ratio (Fig. 7c), which reveals a better differentiation between the cultivars and the basal stem sections than the IAA/CKt ratio (Fig. 7b). Moreover, the mean values calculated for the periods 0–24 hpe and 0–48 hpe showed the best correspondence with the cultivar dependence and topography of AR formation (Fig. 5a–e) for the IAA/CKtABA ratio (Fig. 7d). The data strongly suggest that the ratios of the inducer IAA to the inhibitory cytokinins and ABA attained during the induction phase determine the number of roots, as influenced by the two cultivars and the distance to the wounding site of the cuttings. It can be expected that this ratio was strongly shifted toward higher values by IAA application, allowing similar AR formation in both cultivars (Fig. 1).

The inhibitory activity and auxin antagonism of ABA in AR formation in *H. macrophylla* are supported by findings from other plant species. In tobacco cells, ABA abolished auxin-induced telomerase activity, which in plants shows a strong correlation with the capacity for cell division (Yang et al. 2002). Furthermore, exogenous application of ABA to Arabidopsis seedlings reduced AR formation on hypocotyls in a dose-dependent manner, whereas two ABA biosynthesis mutants and several ABA signaling mutants produced a higher number of ARs (Zeng et al. 2021). Antagonistic relationships between ABA and auxin have been reported for several processes of plant development and growth, such as hypocotyl elongation, lateral root development, root hair expansion, and plant stress responses (Emenecker and Strader 2020; Lei et al. 2023; Ortiz-García et al. 2023). Depending on the specific process, ABA interacts with auxin at the levels of auxin biosynthesis, metabolism, signaling, or even downstream, for example, by counteracting SAUR (Small auxin up RNA)-controlled cell wall acidification. Auxin response factors (ARFs) are important nodes for the crosstalk of auxin with other plant hormones at the level of auxin signaling (Cancé et al. 2022). Most interestingly, it has been shown in Arabidopsis that ABA can induce proteasome-mediated degradation of ARF6 (Li et al. 2020), which functions as a positive regulator of AR formation in the same plant (Gutierrez et al. 2012).

Interestingly, ABA may also act antagonistically to IAA in the regulation of stem elongation. Thus, ABA antagonized IAA-stimulated elongation of tomato hypocotyls by enhancing the expression of an auxin oxidase (Lei et al. 2023). Therefore, the lower ABA levels in the stem base of ‘Caipirinha’ (Fig. 6h) may also explain the stronger IAA response of stem base elongation compared to ‘Clarissa’ (Fig. 2f).

## Conclusion

Although the literature increasingly indicates antagonistic effects of cytokinins and ABA against auxin during AR induction, genetic differences in the rooting competence of cuttings have mostly been attributed to auxin or to auxin/cytokinin ratios. Against this background, the present study provides new insights into the interrelationships among IAA, cytokinins, ABA, carbohydrate metabolism, and AR formation in two contrasting cultivars of *Hydrangea macrophylla*. In this context, we considered the potential roles of specific cytokinins and accounted not only for the intensity but also for the topography of AR formation. The data indicate that free base and riboside cytokinins as well as ABA act as important auxin antagonists during AR induction and thereby limit AR development depending on plant genotype and the distance to the wounding site. Based on these findings, broader screening of additional genotypes and functional analyses of auxin–cytokinin–ABA interrelationships at the molecular and tissue levels can be carried out in the future to further strengthen the relevance of these relationships and to elucidate the underlying mechanisms.

## Supplementary Information

Below is the link to the electronic supplementary material.

Supplementary file1 (PDF 333 KB)

Supplementary file2 (PDF 146 KB)

Supplementary file3 (XLSX 21 KB)

## Data Availability

The original contributions presented in this study are included in the article and Supplementary Materials. Further inquiries can be directed to the corresponding author.

## Abbreviations

AR: Adventitious root
DHZ: Dihydrozeatin
Dpe: Days post excision
Hpe: Hours post excision
IP: Isopentenyladenine
IPR: Isopentenyladenosine
JA: Jasmonic acid
JA-Ile: Jasmonoyl-isoleucine
tZR: *Trans*-zeatin riboside
