# Supplementary material for: Relationships between plant hormones, carbohydrate metabolism and excision-induced adventitious root formation of two contrasting hydrangea cultivars
Source: Planta. 2026 Sep 14;264(4):130. doi: 10.1007/s00425-026-05156-y (PMC13574733; doi:10.1007/s00425-026-05156-y)
Supplement: Supplementary file 1 [file 425_2026_5156_MOESM1_ESM.pdf]

Supplemental Figures to the article in Planta:
Relationships between plant hormones, carbohydrate metabolism and excision-induced adventitious root formation of two contrasting hydrangea cultivars

Uwe Druege, Niklas Mey, Tobias Geißler

Erfurt Research Centre for Horticultural Crops (FGK), Erfurt University of Applied Sciences, Erfurt, Kühnhäuser Str. 101, 99090 Erfurt, Germany

Correspondence: Uwe Druege, uwe.druege@fh-erfurt.de or druege@baum.uni-hannover.de

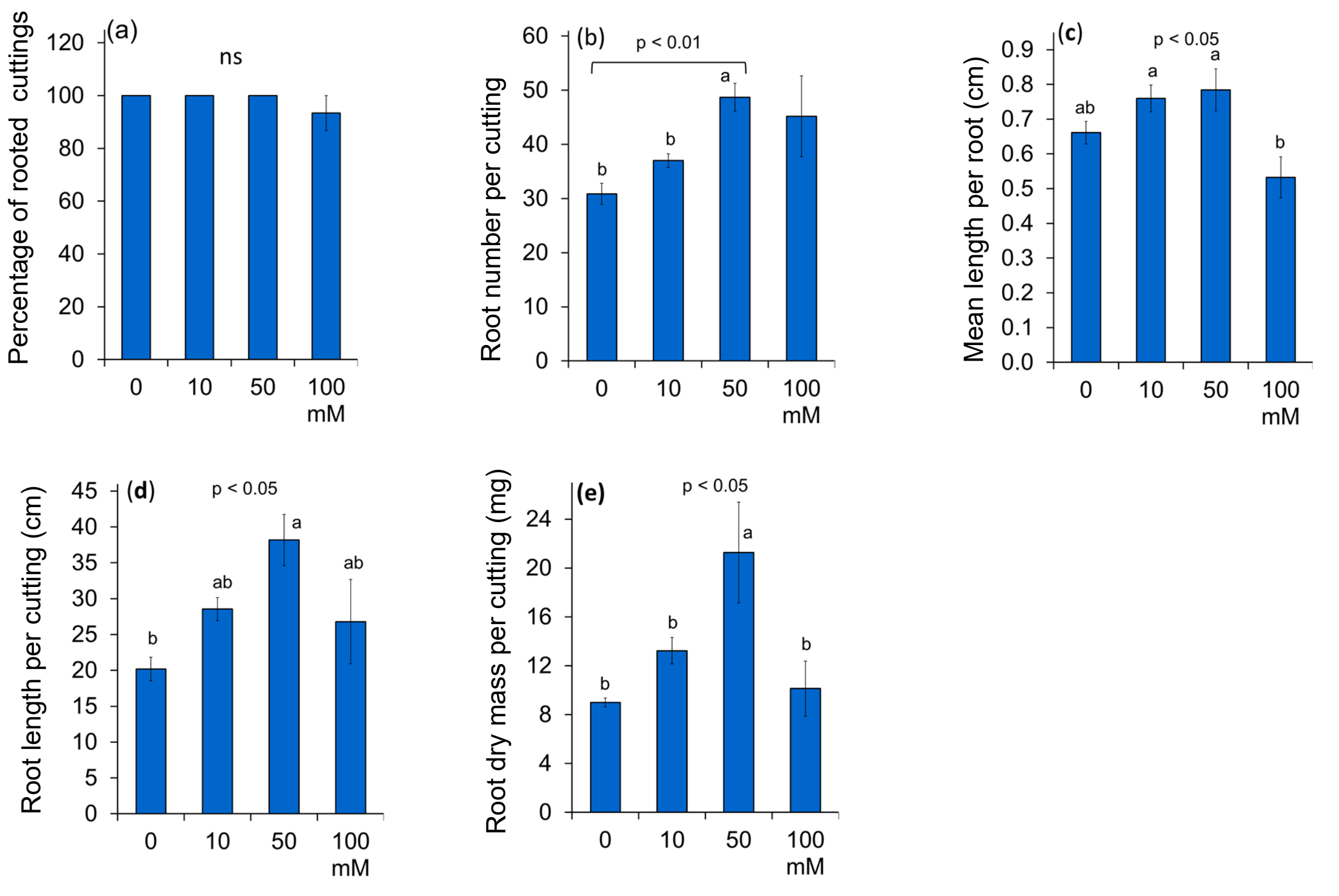

Fig. S1 Effect of pulsed indole-3-acetic acid (IAA) at different concentrations on the percentage of rooted cuttings (**a**), number (**b**), mean length (**c**), total length (**d**) and dry mass (**e**) of adventitious roots of *H. macrophylla* 'Caipirinha'. Determined at 21 days post excision. Mean values ± SE. *P* values indicate the significance of the auxin effect. Different letters mark significant differences based on ANOVA and the Newman–Keuls test ($P < 0.05$). ($n = 3$, each n consisting of data from 10 cuttings)

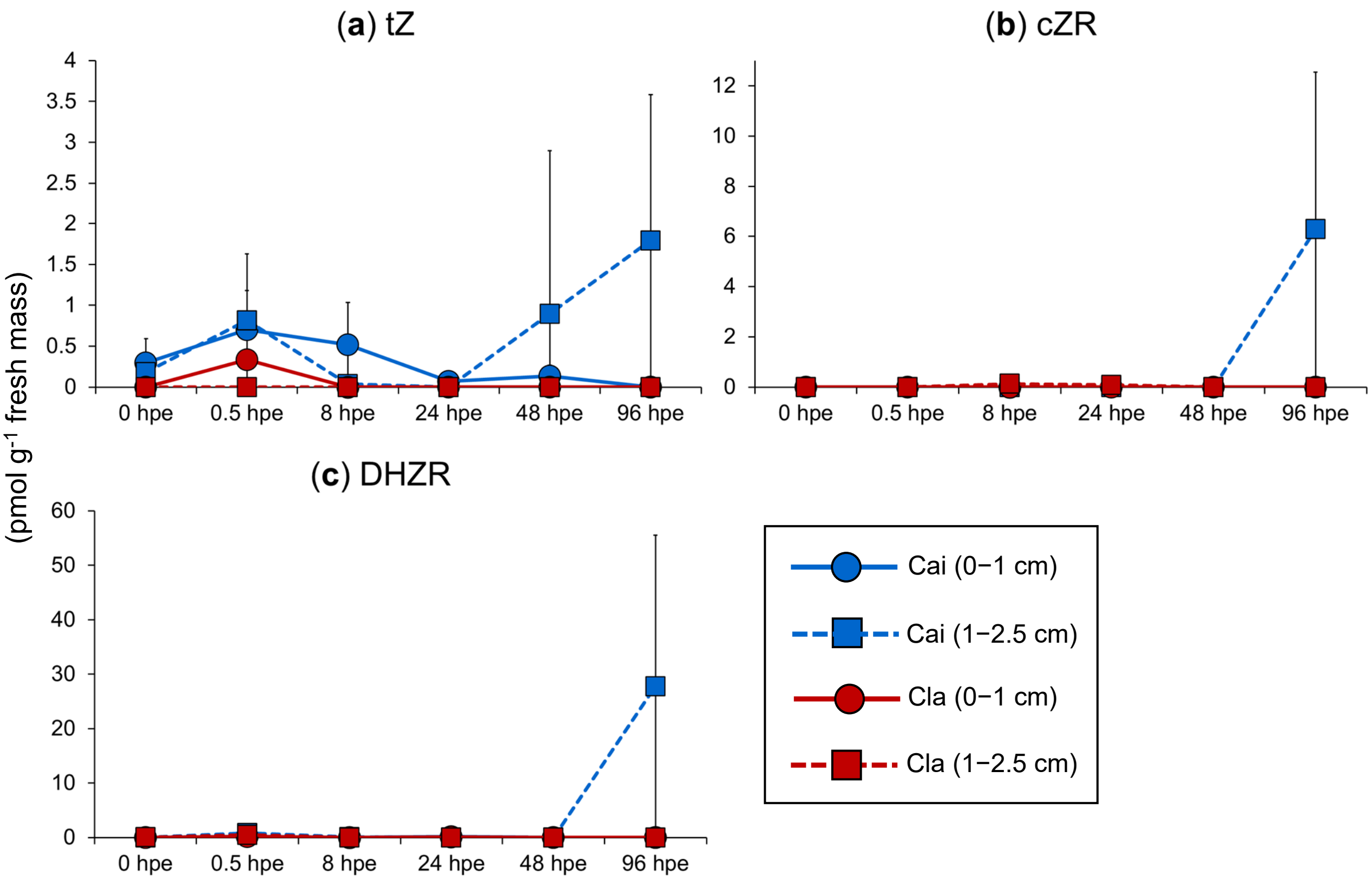

Fig. S2 Effects of cultivar and basal stem section on the dynamics of tZ *(trans-zeatin*) (**a**), cZR (*cis*-zeatin riboside) (**b**), and DHZR (dihydrozeatin riboside) (**c**) during the first 4 days after excision of *H. macrophylla* cuttings. Mean values ± SE (*n* = 5). Cai, 'Caipirinha'; Cla, 'Clarissa'. Hpe, hours post excision. Experiment 3.
