# Supplementary material for: Relationships between plant hormones, carbohydrate metabolism and excision-induced adventitious root formation of two contrasting hydrangea cultivars
Source: Planta. 2026 Sep 14;264(4):130. doi: 10.1007/s00425-026-05156-y (PMC13574733; doi:10.1007/s00425-026-05156-y)
Supplement: Supplementary file 2 [file 425_2026_5156_MOESM2_ESM.pdf]

Supplemental Tables related to the article in Planta:

Relationships between plant hormones, carbohydrate metabolism and excision-induced adventitious root formation of two contrasting hydrangea cultivars

Uwe Druege, Niklas Mey, Tobias Geißler

Erfurt Research Centre for Horticultural Crops (FGK), Erfurt University of Applied Sciences, Erfurt, Kühnhäuser Str. 101, 99090 Erfurt, Germany

Correspondence: Uwe Druege, uwe.druege@fh-erfurt.de or druege@baum.uni-hannover.de

Table S1 Two-factor ANOVA results of the effects of cultivar (C) and IAA pulse application (A) on different rooting parameters of *H. macrophylla*.

	Experiment 1			Experiment 2		
Parameter	C	A	C × A	C	A	C × A
Percentage of rooted cuttings	**	*	**	ns	ns	ns
Number of roots per cutting	ns	****	*	ns	****	ns
Mean length per root (cm)	*	ns	ns	****	**	**
Root length per cutting (cm)	ns	****	ns	ns	**	ns
Root dry mass per cutting (mg)	ns	*	ns	ns	ns	ns
Length of rooting zone (cm)	****	****	ns	**	****	ns
Length of stem base (cm)	**	***	ns			
Length increase of stem base (mm)				****	****	**

*, **, ***, **** significant at $P < 0.05$, 0.01, 0.001, 0.0001, respectively ($n = 4$ in experiment 1, $n = 3$ in experiment 2, each n consisting of data from 10 cuttings); ns, non-significant

Table S2 Three-factor ANOVA results of the effects of cultivar (C), basal stem section (S) and IAA pulse application (A) on concentrations and ratios of carbohydrates in cuttings of *H. macrophylla,* analysed at seven days post excision and planting ($n = 8$).

Carbohydrate concentrations or ratios	C	S	A	C × S	C × A	S × A	C × S × A
Glucose	****	****	****	*	**	ns	ns
Fructose	ns	****	****	ns	*	ns	ns
Sucrose	****	ns	****	ns	**	ns	ns
Total sugars	*	****	****	ns	**	ns	ns
Starch	****	ns	****	ns	***	ns	ns
TNC	**	****	****	ns	***	ns	ns
Hexose/sucrose ratio	***	*	ns	ns	ns	ns	ns
Total sugar ratio (0 – 1 cm) / (1 – 2.5 cm)	*	nd	*	nd	ns	nd	nd

*, **, ***, **** significant at $P < 0.05$, 0.01, 0.001, 0.0001, respectively; ns, non-significant; nd, not determined; total sugars = glucose + fructose + sucrose; TNC, total non-structural carbohydrates = total sugars + starch

Table S3 Three-factor ANOVA results of the effects of cultivar (C), basal stem section (S), and time after planting (T) on concentrations and ratios of plant hormones in cuttings of *H. macrophylla* ($n = 5$).

Hormone	C	S	T	C × S	C × T	S × T	C × S × T
JA	ns	****	****	ns	ns	****	ns
JA-Ile	ns	****	****	ns	ns	****	ns
IAA	ns	ns	****	ns	ns	ns	ns
tZ	*	ns	ns	ns	ns	ns	ns
tZR	ns	*	****	ns	ns	ns	ns
cZ	nt	nt	nt	nt	nt	nt	nt
cZR	ns	ns	ns	ns	ns	ns	ns
IP	**	ns	ns	ns	ns	ns	ns
IPR	****	****	****	ns	ns	ns	ns
DHZ	****	ns	ns	ns	ns	ns	ns
DHZR	ns	ns	ns	ns	ns	ns	ns
ABA	****	*	****	ns	***	ns	ns
IAA/CKb	****	ns	ns	ns	ns	ns	ns
IAA/CKt	****	***	****	ns	ns	ns	ns
IAA/CKtABA	***	****	****	ns	**	**	ns

*, **, ***, **** significant at $P < 0.05$, 0.01, 0.001, 0.0001, respectively; ns, non-significant; nt, not detected. JA, jasmonic acid, JA-Ile, jasmonoyl isoleucine; IAA, indole-3-acetic acid; tZ, *trans*-zeatin; tZR, *trans*-zeatin riboside; cZ, *cis*-zeatin; cZR, *cis*-zeatin riboside; IP, isopentenyladenine; IPR, isopentenyladenine riboside; DHZ, dihydrozeatin; DHZR, dihydrozeatin riboside; ABA, abscisic acid; CKb, cytokinin bases as sum of tZ, IP and DHZ; CKt, total cytokinins as sum of CK bases, tZR, cZR, IPR, DHZR; CKtABA, sum of all detected CKs and ABA.

Table S4 Correlation matrix of plant hormones in basal stem of *H. macrophylla*. Pooled data from the cvs 'Caipirinha' and 'Clarissa', the 0–1 cm and 1–2.5 cm basal stem sections, and the time points 0, 0.5, 8, 24, 48 and 96 hours post excision. Red letters mark significant correlations (t-test, $P < 0.05$, $n = 120$).

Plant hormone concentration or ratio	JA	JA-Ile	IAA	tZR	IP	IPR	DHZ	ABA	IAA/ CKb	IAA/ CKt	IAA/ CKtABA
JA	1.000	0.932	0.202	0.425	0.173	0.142	0.109	0.437	0.018	-0.209	-0.273
JA-Ile	0.932	1.000	0.174	0.434	0.105	0.162	0.136	0.368	0.018	-0.213	-0.213
IAA	0.202	0.174	1.000	0.339	0.138	0.211	0.073	0.176	0.389	0.342	0.184
tZR	0.425	0.434	0.339	1.000	0.055	0.418	-0.087	0.589	0.213	-0.296	-0.332
IP	0.173	0.105	0.138	0.055	1.000	0.235	0.427	0.205	-0.413	-0.408	-0.259
IPR	0.142	0.162	0.211	0.418	0.235	1.000	0.255	0.610	-0.074	-0.381	-0.319
DHZ	0.109	0.136	0.073	-0.087	0.427	0.255	1.000	0.122	-0.610	-0.476	-0.142
ABA	0.437	0.368	0.176	0.589	0.205	0.610	0.122	1.000	-0.040	-0.431	-0.516
IAA/CKb	0.018	0.018	0.389	0.213	-0.413	-0.074	-0.610	-0.040	1.000	0.558	0.126
IAA/CKt	-0.209	-0.213	0.342	-0.296	-0.408	-0.381	-0.476	-0.431	0.558	1.000	0.735
IAA/CKtABA	-0.273	-0.213	0.184	-0.332	-0.259	-0.319	-0.142	-0.516	0.126	0.735	1.000

JA, jasmonic acid; JA-Ile, jasmonoyl isoleucine; IAA, indole-3-acetic acid; tZ, *trans*-zeatin; tZR, *trans*-zeatin riboside; cZR, *cis*-zeatin riboside; IP, isopentenyladenine; IPR, isopentenyladenine riboside; DHZ, dihydrozeatin; DHZR, dihydrozeatin riboside; ABA, abscisic acid; CKb, cytokinin bases as sum of tZ, IP and DHZ; CKt, total cytokinins as sum of CKb, tZR, cZR, IPR, and DHZR; CKtABA as sum of all detected CKs and ABA
